# Multiregulatory hydrogel supramolecular nanomedicine for reprogramming cartilage homeostasis in osteoarthritis

**DOI:** 10.1016/j.mtbio.2026.103380

**Published:** 2026-06-20

**Authors:** Yibo Ma, Renjie Zhang, Songjie Han, Ziyu Zhuang, Zhenggang Wang, Chenyue Xu, João Conde, Jinyu Li, Qiaoli Zhai, Changjian Chen

**Affiliations:** a Department of Orthopedics, The Second Hospital of Dalian Medical University, Dalian, 116023, China; b Dongzhimen Hospital, Beijing University of Chinese Medicine, Beijing, 100700, China; c School of Medicine, Foshan University, Foshan, 528099, China; d Department of Orthopedics, Tongji Hospital, Tongji Medical College, Huazhong University of Science and Technology, Wuhan, 430030, China; e School of Pharmacy, Nanjing University of Chinese Medicine, Nanjing, 210023, China; f Comprehensive Health Research Centre (CHRC), NOVA Medical School, Faculdade de Ciências Médicas, NMS|FCM, Universidade NOVA de Lisboa, Lisbon, Portugal; g Translational Medicine Center, Zibo Central Hospital Affiliated to Binzhou Medical University, Zibo, 255036, China

**Keywords:** Osteoarthritis, Supramolecular nanoparticle, Hydrogel microsphere, Bioactive core, Delivery carrier

## Abstract

Osteoarthritis (OA) is a multifactorial degenerative joint disorder characterized by intricate interactions among oxidative stress, inflammation, apoptosis, and extracellular matrix (ECM) degradation, ultimately disrupting chondrocyte homeostasis and accelerating cartilage deterioration. Although advanced intra-articular biomaterial systems have improved local therapeutic delivery for OA, the rational integration of a multi-component bioactive core with a multifunctional carrier remains challenging. In this study, a multifunctional nanomedicine, K@BMT@HM, was developed by integrating a supramolecular bioactive core with a functionalized hydrogel microsphere carrier. The K@BMT core, composed of the KRFK peptide, bisdemethoxycurcumin (BDMC), and MnTBAP, exerted multi-dimensional regulatory effects by enhancing antioxidant defense, restoring autophagic flux, and modulating TGF-β-associated signaling, thereby mitigating oxidative stress, inflammation, and apoptosis while favoring ECM anabolic balance in chondrocyte-based assays. However, because TGF-β signaling is highly context- and compartment-dependent, these observations should not be interpreted as evidence that TGF-β activation is uniformly beneficial across the whole joint. Meanwhile, the HM-SCHW carrier, composed of chitosan (CS), sodium alginate (SA), hyaluronic acid (HA), and WYRGRL peptide, provided thermosensitive depot formation, lubrication, cartilage-associated localization/retention, and *in vitro* sustained/ROS-responsive release, thereby supporting localized delivery potential rather than proving WYRGRL-specific cartilage targeting. Moreover, the laser-responsive thermal behavior of K@BMT@HM provided a controllable local heating modality that may support thermosensitive *in situ* retention. Remarkably, the integrated K@BMT@HM, which couples the multi-component regulatory capacity of the K@BMT core with the multifunctional delivery advantages of the HM-SCHW carrier, was validated through a series of *in vitro* and *in vivo* experiments. Collectively, this work presents a rationally designed nanomedicine that integrates bioactive regulation with functionalized delivery to enhance chondroprotection, supporting further preclinical investigation of sustained and multimodal OA intervention. However, because the laser-assisted thermal component was validated only in a rat small-joint model, these findings cannot be directly extrapolated to human OA joints, where deeper articular cartilage and overlying skin, adipose tissue, synovium, and other tissues may substantially limit optical penetration and thermal delivery. In addition, because systematic single-component, two-component, and component-deletion controls were not performed, the present data should be interpreted as validating the integrated K@BMT@HM system as a whole rather than defining the quantitative contribution or indispensability of BDMC, MnTBAP, KRFK, 808-nm laser activation, the CS/SA hydrogel matrix, HA, or WYRGRL.

## Highlights

• K@BMT supramolecular core orchestrates multiple antioxidative, anti-inflammatory, pro-autophagic and anti-apoptotic bioactivities.
• HM-SCHW delivery carrier enables thermosensitive solidification, cartilage targeting, lubrication, and ROS-responsive release.
• Integrated photo-chemotherapy provides combined therapeutic effects for intra-articular osteoarthritis treatment.
• Multimodal imaging and visualization techniques track nanomedicine distribution and efficacy.

## Graphical abstract

**Figure:**
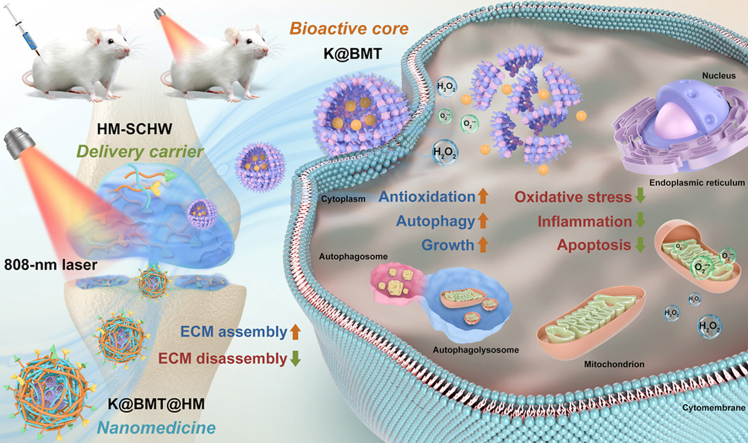

## 1 Introduction

Osteoarthritis (OA) is the most common joint disease in adults. According to the study, about 595 million people were living with OA in 2020 (about 7.6%), representing an increase of roughly 132% since 1990, and the number of cases is projected to continue rising substantially by 2050 [1]. As OA progresses, patients often experience persistent joint pain, deformity, and restricted mobility [2]. At present, there are no effective treatment options for OA, and clinical management mainly relies on conservative strategies such as exercise-based rehabilitation, self-management education, and analgesic medications [1]. Although current pharmacological agents, such as nonsteroidal anti-inflammatory drugs (NSAIDs), can temporarily mitigate pain and inflammation, they are unable to arrest disease progression or restore the structural integrity of the joint [2]. Once OA progresses to its terminal stage, joint replacement and other surgical procedures often constitute the sole remaining means of treatment [2]. Accordingly, there is an urgent need to develop novel therapeutic strategies that effectively delay or even reverse the progression of OA.

The hallmark pathological alteration of OA is the progressive degenerative deterioration of articular cartilage [3]. Chondrocytes, the sole resident cells in cartilage, are responsible for maintaining extracellular matrix (ECM) homeostasis, with type II collagen and aggrecan as major structural components [4]. This ECM not only provides the mechanical foundation for load dissipation but also serves as a signaling microenvironment that supports chondrocyte survival and phenotypic stability [5]. During OA progression, mechanical overload and inflammatory cues within the joint microenvironment converge to drive a sustained catabolic program, characterized by the overproduction of matrix-degrading enzymes such as a disintegrin and metalloproteinase with thrombospondin motifs (ADAMTS) and matrix metalloproteinases (MMPs), which directly accelerates cartilage matrix loss [6]. Mechanistically, chronic inflammation and oxidative stress form a self-amplifying loop: inflammatory cytokines promote excessive reactive oxygen species (ROS) generation, while ROS further potentiates inflammatory signaling, leading to persistent activation of stress-responsive pathways (e.g., NF-κB, TNF, MAPK, PI3K-AKT, and JAK/STAT) [7]. This sustained inflammatory–oxidative milieu not only reinforces ADAMTS/MMP expression but also compromises endogenous protective programs, including attenuation of the NRF2–ARE antioxidant axis [8]. As oxidative stress escalates beyond the buffering capacity of antioxidant enzymes, lipid peroxidation and organelle injury become prominent, particularly endoplasmic reticulum (ER) stress and mitochondrial dysfunction [9,10]. In parallel, impaired autophagy in chondrocytes limits the clearance of damaged organelles and proteotoxic cargos, thereby exacerbating intracellular stress and ultimately promoting apoptosis [11,12]. TGF-β signaling has been implicated in chondrocyte phenotype regulation and matrix metabolism; however, its biological consequences in OA are context-dependent and may vary by tissue compartment, disease stage, receptor usage, and local mechanical or inflammatory cues [13]. Therefore, restoring chondrocyte homeostasis requires coordinated interventions to (1) reinforce antioxidant defenses to maintain redox balance; (2) reactivate autophagy to preserve organelle quality control; (3) temper inflammatory signaling to reduce catabolic enzyme induction; and (4) cautiously modulate TGF-β–associated signaling in a manner compatible with cartilage anabolic balance while avoiding overinterpretation of whole-joint benefits [14].

In recent years, biomaterials have been widely applied in the treatment of OA and have attracted increasing attention [15]. A variety of three-dimensional biodegradable scaffolds, including injectable hydrogels, cell-derived membranes, and liposomes, have been developed as intra-articular nano-delivery systems designed to achieve targeted therapeutic effects [16,17]. In particular, hydrogel microsphere-based systems have recently been used to construct lubrication barriers and regulate inflammation-related communication during dynamic tissue repair [18]. Despite the crucial role of biomaterial scaffolds in joint repair and drug delivery, several limitations remain. On the one hand, natural hydrogels, owing to their inherently loose molecular architecture, are often incapable of providing adequate load-bearing support and are prone to rapid degradation *in vivo*, thereby limiting their long-term stability within the joint cavity [19]. Although synthetic polymer-based scaffolds exhibit superior mechanical strength, their surfaces lack cell-adhesive and bio-recognition motifs, and their degradation may generate acidic by-products that trigger local inflammation or perturb cellular functions [20]. Furthermore, certain liposomal carriers are highly sensitive to subtle environmental changes—such as shear stress, temperature fluctuations, or alterations in solution composition—rendering them structurally unstable [21]. On the other hand, numerous delivery carriers suffer from insufficient targetability and responsiveness, thereby restricting their ability to achieve accurate modulation of intra- and extracellular microenvironments [22,23]. Among stimulus-responsive strategies, laser-assisted thermal intervention provides an additional regulatory modality for OA therapy. Under near-infrared irradiation, thermally responsive nanomaterials can generate controllable local heating, which has been applied in preclinical OA models to promote on-demand drug release, enhance local therapeutic responses, and modulate oxidative or inflammatory microenvironments [24,25]. Therefore, considering the complexity of OA pathogenesis and the limitations of biomaterials, effective nanomedicines should integrate a potent bioactive core with an accurate delivery carrier, thereby enabling targeted delivery to cartilage tissue and regulation of chondrocytes, and ultimately exerting comprehensive effects that can delay OA progression. Nevertheless, the biological contribution of laser-assisted heating should be distinguished from nonspecific mild hyperthermia, altered release, uptake changes, or stress-response pathways using mechanism-specific controls. From a translational perspective, the feasibility of this laser-assisted strategy in human OA joints remains unproven, because human articular cartilage is deep-seated and shielded by skin, adipose tissue, synovium, and other periarticular tissues. Therefore, efficacy observed in small-animal models should not be directly extrapolated to human OA without ex vivo thick-tissue penetration studies, large-joint validation, and optical-thermal modeling.

**Scheme 1:**
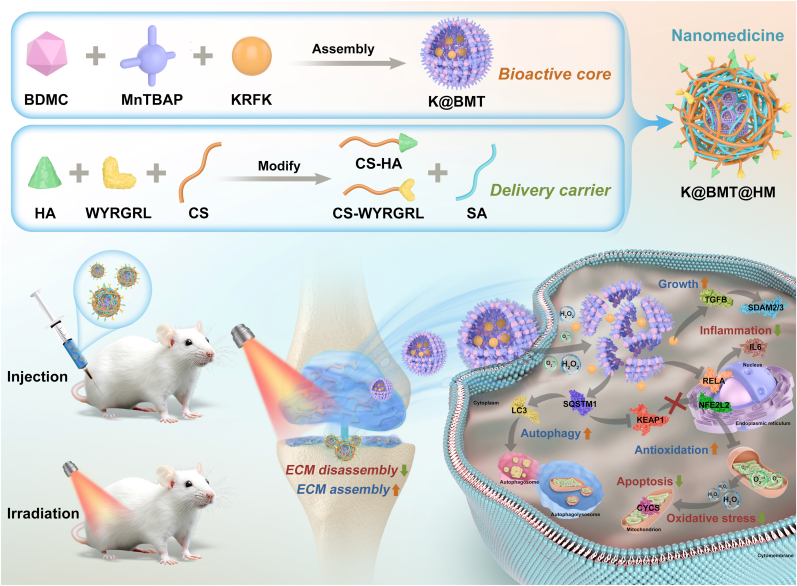
Schematic illustration of the fabrication process and regulatory mechanism of the K@BMT@HM, which serves as a nanomedicine for OA therapy.

In light of the aforementioned considerations, this work reports the design and development of a nanomedicine. Guided by the major pathological axes of OA, three compounds, bisdemethoxycurcumin (BDMC), KRFK, and MnTBAP, were selected through compound-library screening and self-assembled into supramolecular nanoparticles (KRFK@BDMC-MnTBAP, K@BMT), constituting the bioactive core of the nanomedicine. Briefly, KRFK was incorporated to modulate TGF-β-associated signaling observed in chondrocyte assays, BDMC was selected for its pleiotropic regulation of oxidative stress, inflammatory activation, apoptosis, senescence, and autophagy-related cellular dysfunction, and MnTBAP was introduced to provide SOD-mimetic ROS-scavenging activity and laser-responsive thermal behavior. Because TGF-β signaling may exert divergent cartilage, synovial, periarticular, and subchondral effects, the present design should be interpreted as exploring TGF-β-associated regulatory changes rather than establishing joint-wide beneficial TGF-β activation. Chitosan (CS) was modified with hyaluronic acid (HA) and WYRGRL peptide, and subsequently co-assembled with sodium alginate (SA) into thermosensitive hydrogel microspheres (HM), labeled as HM-SA-CS-HA-WYRGRL (HM-SCHW), which served as the delivery carrier of the nanomedicine. In the present study, the WYRGRL-containing carrier is described as supporting cartilage-associated localization/retention, whereas WYRGRL-specific targeting requires further control validation. By encapsulating the K@BMT core into the HM-SCHW carrier, a composite nanomedicine named K@BMT@HM was established. Nevertheless, the regulatory effects of this nanomedicine on chondrocytes and its cartilage-protective potential remain to be elucidated. Thus, it is necessary to explore both the therapeutic efficacy and pharmacological mechanisms of K@BMT@HM for OA therapy. Herein, the K@BMT core was investigated through cellular experiments to evaluate its feasibility and regulatory mechanisms for OA therapy, whereas K@BMT@HM was further evaluated in a rat OA model to assess its therapeutic efficacy (Scheme 1). With supportive evidence from both *in vitro* and *in vivo* evaluations, K@BMT@HM may constitute a preclinical proof-of-concept strategy for OA intervention. The current evidence supports feasibility in cellular systems and a rat OA model, but it does not establish optical penetration, heating controllability, or therapeutic applicability in human joints. The current experimental design was intended to evaluate the feasibility and therapeutic performance of this integrated platform; it was not designed to deconvolute the necessity, sufficiency, or quantitative contribution of each individual component or module.

## 2 Materials and methods

### 2.1 Model establishment

Advanced glycation end products (AGEs) (50 μg/mL), interleukin-1 beta (IL1B) (10 ng/mL), and tumor necrosis factor-alpha (TNFA) (20 ng/mL) treatments were applied to induce OA chondrocyte and cartilage phenotypes, whereas anterior cruciate ligament transection (ACLT) and destabilization of the medial meniscus (DMM) were performed to establish OA rat models. All zoological experiments were permitted by the ethics committee of Nanjing University of Chinese Medicine (No. 202411A023). Detailed descriptions of the material, cellular, and zoological experiments are provided in the Supplementary data.

### 2.2 Statistical analysis

All *in vitro* experiments in this study were independently repeated three times, whereas each group in the *in vivo* experiments consisted of five rats. Data were analyzed using SPSS 26.0 and are presented as the mean ± standard deviation (X ± S). Before statistical comparisons, data normality was evaluated using the Shapiro–Wilk test, and homogeneity of variance was assessed using Levene's test. For comparisons between two groups, statistical significance was determined using two-tailed Student's t-tests. For comparisons among multiple groups, one-way ANOVA was performed, followed by Tukey's post hoc test for multiple comparisons. *P* < 0.05 was considered statistically significant.

## 3 Result and disscussion

### 3.1 Design and characterization of K@BMT core

As mentioned above, the bioactive core should be endowed with pharmacological activity that regulates diverse intracellular signaling cascades in chondrocytes. As a result of screening, three compounds were identified as candidate OA-modulating components for constructing the K@BMT core. KRFK, identified as a peptide fragment originating from thrombospondin-1 (TSP-1) [26], interacts with the LSKL site of LAP, leading to activation of latent TGF-β and consequent changes in downstream pathways [27]. Nevertheless, because TGF-β signaling can exert compartment-specific and disease-stage-dependent effects, including potentially undesirable profibrotic responses outside cartilage, KRFK-related TGF-β pathway activation should be interpreted cautiously in this study. BDMC, a natural product derived from *Curcuma longa* L., exhibits diverse biological functions, notably antioxidant and anti-inflammatory activities mediated via activation of the NFE2L2-ARE axis and suppression of pro-inflammatory signaling such as NF-κB [28,29], as well as anti-aging [30], anti-apoptotic [31], and pro-autophagic effects [32]. MnTBAP, a manganese porphyrin bearing four para-carboxyphenyl substituents and a central Mn(III) ion, has been utilized as a metal–organic framework for nanoparticle fabrication [33]. Of note, MnTBAP mimics the catalytic activity of superoxide dismutase (SOD), thereby efficiently scavenging superoxide anion (O2•^-^) [34,35]. It can convert near-infrared irradiation into heat and thereby provides laser-responsive thermal properties, although the downstream biological contribution of this heating requires cautious interpretation in the absence of temperature-matched and component-specific controls [24,36]. Therefore, based on the distinct biological activities of these compounds, the K@BMT core was constructed as the bioactive component of the nanomedicine to support multi-level regulation of OA-related chondrocyte dysfunction. Accordingly, although the selected components were incorporated on the basis of their reported functional properties and the module-level comparisons provide useful supportive evidence, the present study does not establish the individual indispensability or relative contribution of KRFK, BDMC, MnTBAP, or laser-responsive activation within the composite system.

**Fig. 1:**
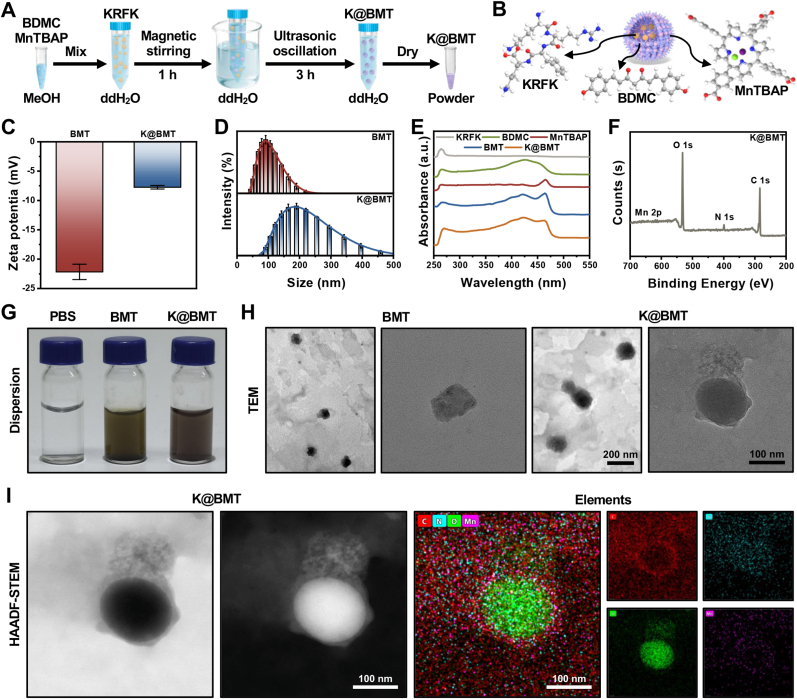
Synthesis and characterization of BMT and K@BMT core. **(A)** Assembly process illustration of K@BMT core. **(B)** Chemical structures of KRFK, BDMC, and MnTBAP monomers and hierarchical organization of K@BMT core. **(C)** Zeta potential of BMT and K@BMT core. **(D)** Particle sizes of BMT and K@BMT core. **(E)** UV absorbance of KRFK, BDMC, MnTBAP, BMT, and K@BMT core. **(F)** XPS spectrum of K@BMT core for C 1s, N 1s, O 1s, and Mn 2p. **(G)** Dispersion of BMT and K@BMT core in PBS. **(H)** LV-TEM and HR-TEM images of BMT and K@BMT core. **(I)** HAADF-STEM and corresponding elemental mappings of C, N, O, and Mn elements in K@BMT core. All data are presented as the mean ± SD (n = 3).

The assembly scheme of the K@BMT core (Fig. 1A), along with the chemical structure and hierarchical arrangement of KRFK, BDMC, and MnTBAP monomers (Fig. 1B, Fig. S1A), is presented in the figure. After design and synthesis, BDMC-MnTBAP (BMT) and K@BMT were systematically characterized to assess their suitability as bioactive cores. As depicted in the figure, the zeta potential measurements revealed values of −22.17 mV for the BMT core and −7.743 mV for the K@BMT core (Fig. 1C), while the corresponding particle size analysis demonstrated diameters of approximately 90 nm and 180 nm, respectively (Fig. 1D). The observed changes in the Ultraviolet (UV) absorption peak positions of KRFK, BDMC, MnTBAP, BMT, and K@BMT core further confirmed the progression of core synthesis (Fig. 1E). Furthermore, X-ray photoelectron spectroscopy (XPS) analysis was employed to investigate the valence states of elements in the K@BMT core (Fig. 1F). Next, the K@BMT core was visualized to characterize its morphology. The self-assembled BMT and K@BMT cores maintained stable suspensions in phosphate-buffered saline (PBS) (Fig. 1G). Furthermore, low-voltage transmission electron microscopy (LV-TEM) and high-resolution TEM (HR-TEM) imaging provided morphological evidence for the BMT and K@BMT core (Fig. 1H), and elemental mappings obtained by high-angle annular dark-field scanning transmission electron microscope (HAADF-STEM) confirmed the presence of C, N, O, and Mn in the K@BMT core (Fig. 1I).

Based on this design rationale, the K@BMT core was fabricated through magnetic stirring combined with ultrasonic oscillation. A series of characterizations, including TEM and HAADF-STEM for morphology and elemental distribution, zeta potential and particle size measurements, UV absorption peak shifts, and XPS analysis of elemental valence states, collectively demonstrated that KRFK, BDMC, and MnTBAP monomers were successfully assembled into a stable K@BMT core under optimized conditions.

### 3.2 Performance validation of K@BMT core

Having confirmed the successful assembly and structural characteristics of the K@BMT core, its functional performance was further examined with emphasis on ROS responsiveness, colloidal stability, and laser-responsive thermal properties, which are relevant to the proposed laser-assisted delivery strategy and subsequent therapeutic application.

**Fig. 2:**
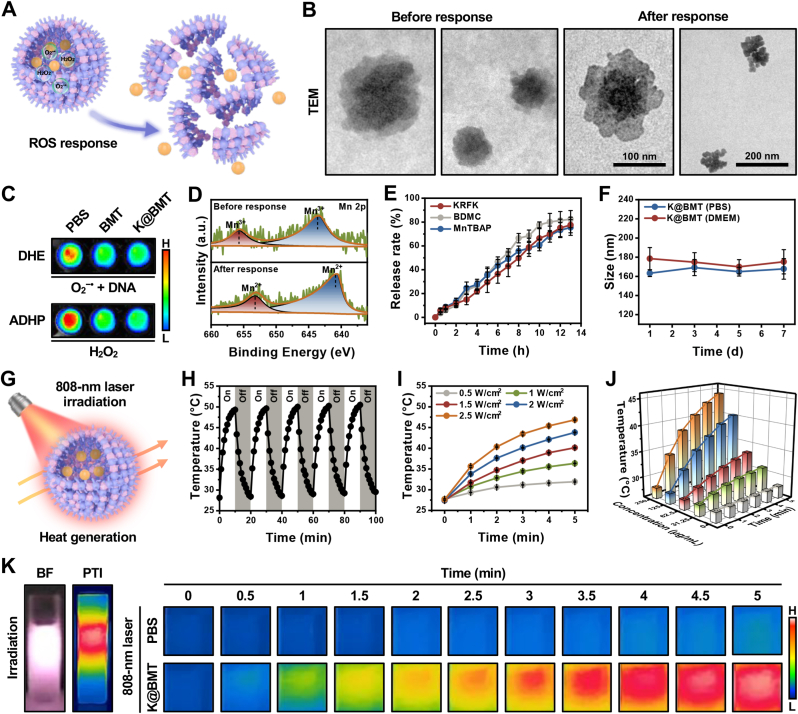
Performance validation of BMT and K@BMT core. (A) ROS responsiveness of K@BMT core. (B) LV-TEM images of K@BMT core before and after ROS response. (C) FI images for O2•^-^ and H2O2 with DHE and ADHP probes. (D) XPS spectrum of K@BMT core before or after ROS response for Mn 2p. (E) Release rate of KRFK, BDMC, and MnTBAP from K@BMT core after ROS response. (F) Stability test of K@BMT core in PBS and DMEM. (G) Laser-responsive thermal effect of K@BMT core. (H) Temperature-change curves of K@BMT core under five repeated “heating/cooling” 808-nm laser. (I) Temperature-change curves of K@BMT core (250 μg/mL) under 808-nm laser with different power density (0.5, 1, 1.5, 2, and 2.5 W/cm2) for 5 min. (J) Temperature-change of K@BMT core with different concentrations (0, 31.25, 62.5, 125, and 250 μg/mL) under 808-nm laser (2 W/cm2) for 5 min. (K) Panoramic views of bright-field (BF) and PTI with K@BMT core under 808-nm laser as well as PTI images of PBS and K@BMT core (125 μg/mL) under 808-nm laser (2 W/cm^2^) for 5 min. All data are presented as the mean ± SD (n = 3).

Considering the excessive ROS accumulation in osteoarthritic microenvironments and the reported antioxidant effects of MnTBAP through Mn(III)-mediated redox cycling, the ROS responsiveness of the K@BMT core was first evaluated under simulated oxidative stress conditions (Fig. 2A). TEM observation revealed marked morphological changes of K@BMT after ROS exposure, suggesting ROS-triggered structural disassembly of the supramolecular core (Fig. 2B). To further verify its ROS-scavenging capability, DHE and ADHP probes were used to detect O_2_•^-^ and hydrogen peroxide (H_2_O_2_), respectively. Compared with PBS, both BMT and K@BMT markedly reduced the fluorescence signals corresponding to O_2_•^-^ and H_2_O_2_, indicating that the MnTBAP-containing core retained ROS-eliminating activity after supramolecular assembly (Fig. 2C). This behavior is consistent with the reported redox-cycling activity of Mn porphyrin-based SOD mimetics, which can catalytically regulate superoxide-related oxidative stress. To clarify the redox change of the Mn center, XPS analysis was performed before and after ROS response [37]. The Mn 2p spectrum showed that the valence state of Mn shifted from Mn^3+^-dominant signals before response to Mn^2+^-related signals after response (Fig. 2D), supporting the involvement of Mn-centered redox conversion during ROS scavenging. Moreover, the release profiles of the three active components, KRFK, BDMC, and MnTBAP, were quantitatively evaluated under ROS-stimulated conditions. All three components exhibited time-dependent release after ROS exposure, with their cumulative release levels reaching approximately 70–80% within 12–13 h (Fig. 2E). These release kinetic curves provided quantitative evidence that ROS exposure induced structural transformation of K@BMT and promoted the time-dependent release of its bioactive components, thereby supporting its responsiveness to oxidative OA microenvironments. After confirming ROS responsiveness, the stability of K@BMT was examined in physiologically relevant media. The hydrodynamic size of K@BMT remained relatively stable in both PBS and DMEM over 7 days, without obvious aggregation or size fluctuation (Fig. 2F). This result suggests that the supramolecular core possesses sufficient dispersion stability under storage and biological application conditions.

Since MnTBAP has been reported to possess photothermal characteristics, thermal imaging was employed to monitor temperature changes under 808-nm laser irradiation and to determine whether the assembled K@BMT core retained laser-responsive thermal conversion (Fig. 2G). The thermal cycling behavior of the K@BMT core was then evaluated to assess the stability of this laser-responsive temperature elevation. After five heating/cooling cycles, no obvious changes were observed in the maximum temperature, heating rate, or cooling rate, indicating stable thermal responsiveness of the K@BMT core (Fig. 2H). In addition, the laser irradiation power density and drug concentration are key parameters [38]. The results showed that the heating rate and final temperature rise were positively correlated with both power density (Fig. 2I) and concentration (Fig. 2J), indicating that the thermal behavior of the K@BMT core was dependent on these variables. Notably, the K@BMT core at a concentration of 250 μg/mL under 2.5 W/cm^2^ laser power induced a rapid temperature elevation from approximately 28 °C to 47 °C within 5 min.

As a prerequisite for subsequent cellular experiments, the cells employed in this study were authenticated as chondrocytes based on positive Safranin O (SO) staining (Fig. S2A). As described earlier, the K@BMT core showed clear laser-responsive thermal conversion; nevertheless, temperatures exceeding 43 °C in the pericellular environment can significantly disrupt metabolic function and provoke adverse biological processes, such as inflammation and apoptosis [25]. According to the results above, it was observed that the K@BMT core at 250 μg/mL under 1.5 W/cm^2^laser irradiation, as well as that at 125 μg/mL under 2 W/cm^2^, was able to raise the temperature from 28 °C to 41 °C within approximately 5 min. In selecting the irradiation condition for further experiments, both controllable mild heating and biological safety were considered, without assuming that temperature elevation alone accounts for the subsequent biological effects. To ensure safe experimental conditions, it was considered essential to examine chondrocyte viability under varying concentrations of the K@BMT core, as well as the K@BMT core under 808-nm laser. According to cytotoxicity assay results, the K@BMT core was biocompatible within the concentration range of 0–200 μg/mL, and 808-nm laser irradiation (2 W/cm^2^) did not significantly affect chondrocyte viability (Fig. S2B). Considering both thermal responsiveness and biological safety, 125 μg/mL K@BMT combined with 808-nm laser irradiation at 2 W/cm^2^ was ultimately selected as the experimental irradiation condition for subsequent experiments. As shown in the figure, photothermal imaging (PTI) and real-time monitoring revealed that the liquid temperature in the K@BMT core group rose steadily within 5 min compared with the PBS group under the selected irradiation condition (Fig. 2K).

Collectively, these results demonstrated that the K@BMT core possesses ROS-responsive activity, colloidal stability, and controllable laser-responsive thermal properties. The stable hydrodynamic size of K@BMT in PBS and DMEM over 7 days indicated favorable colloidal stability, supporting its suitability for subsequent biological applications. Under oxidative conditions, the K@BMT core not only scavenged O2•^-^ and H2O2 through Mn-centered redox conversion, but also underwent structural disassembly accompanied by the release of KRFK, BDMC, and MnTBAP. This ROS-triggered response provides a basis for microenvironment-adaptive release of bioactive components in ROS-enriched OA lesions, where excessive ROS is closely associated with oxidative stress and cartilage degeneration. In addition, the 808-nm laser-responsive temperature elevation of K@BMT may provide mild thermal input for the thermosensitive HM carrier and may contribute to the observed laser-assisted effects. However, these physicochemical data alone cannot distinguish specific photothermal activation from nonspecific mild hyperthermia or downstream effects such as altered release, cellular uptake, or stress-response signaling. Therefore, the K@BMT core provides a physicochemical foundation for subsequent chondrocyte homeostasis modulation and intra-articular OA therapy, while the biological mechanism of laser assistance should be interpreted cautiously.

### 3.3 Exploration of biologic functions regulated by K@BMT core

Building on the previous design, characterization, and performance validation of the K@BMT core, a comprehensive investigation was undertaken to evaluate whether the K@BMT core, serving as the bioactive core of the nanomedicine, exerts multifaceted regulatory effects on chondrocytes.

**Fig. 3:**
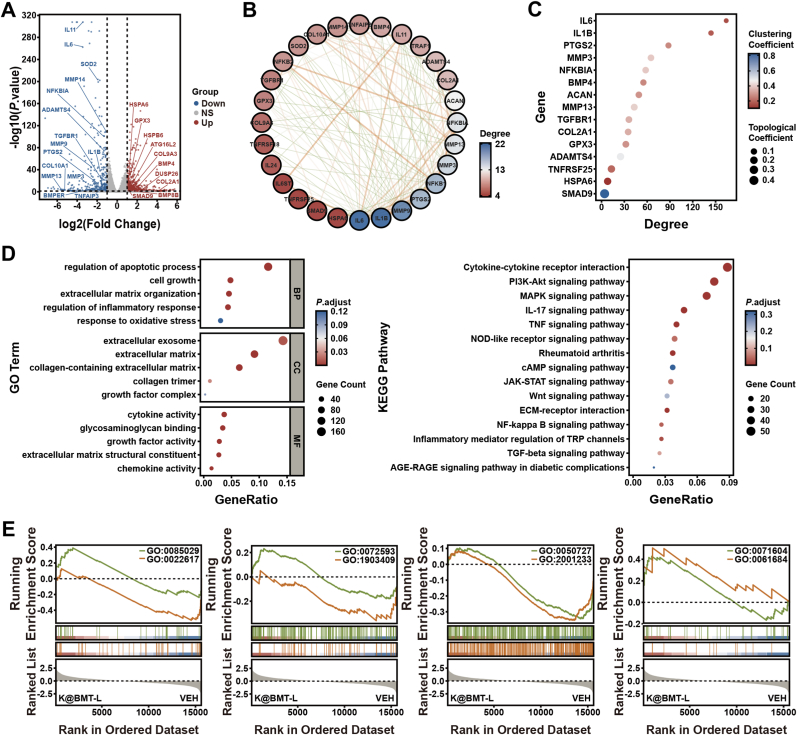
Transcriptome mRNA-Seq and bioinformatics analysis of chondrocytes. **(A)** Volcano plot of DEGs (|Log2FoldChange| > 1, *P* < 0.05). **(B)** PPI network of key genes from DEGs in chondrocytes. **(C)** Topology analysis of DEGs. **(D)** GO and KEGG enrichment analysis of DEGs. **(E)** GSEA based on the biological functions (GO:0085029, GO:0022617, GO:0072593, GO:1903409, GO:0050727, GO:2001233, GO:0071604, and GO:0061684).

Transcriptome mRNA sequencing (mRNA-Seq) was performed on VEH- and K@BMT-L-treated chondrocytes to investigate the gene-expression remodeling associated with K@BMT-L treatment. Bioinformatic analysis identified a total of 1224 differentially expressed genes (DEGs). The volcano plot illustrated the distribution of these DEGs (Fig. 3A), while heatmap visualization (Fig. S2C) and protein-protein interaction (PPI) networks (Fig. S2D) provided an intuitive representation of their diversity and interaction. Additionally, topological analysis of the 1224 DEGs was conducted, and representative DEGs with prominent ranking and statistical significance were visualized (Fig. 3C), while the PPI network was constructed to further illustrate the interaction relationships among these representative DEGs (Fig. 3B). Comprehensive bioinformatic analysis uncovered extensive regulatory information on chondrocyte functions and signaling pathways. Gene Ontology (GO) enrichment analysis demonstrated that K@BMT-L treatment significantly influenced biological processes related to ECM regulation, apoptosis, inflammation, oxidative stress, and growth factors, encompassing 1882 biological process (BP), 172 cellular component (CC), and 223 molecular function (MF) terms (Fig. 3D). Besides, Kyoto Encyclopedia of Genes and Genomes (KEGG) enrichment analysis likewise demonstrated significant pathways related to ECM, inflammation, oxidative stress, and apoptosis, including “Cytokine-cytokine receptor interaction”, “PI3K-Akt signaling pathway”, “MAPK signaling pathway”, “TNF signaling pathway”, “ NF-κB signaling pathway”, “ TGF-β signaling pathway”, and “ECM-receptor interaction” (Fig. 3D). On the basis of GO and KEGG analyses, which identified the primary biological functions and enriched pathways of differentially expressed genes, Gene Set Enrichment Analysis (GSEA) was subsequently applied to explore enrichment trends across the entire transcriptome, thereby enabling a more systematic and comprehensive interpretation. According to GSEA, K@BMT-L treatment significantly regulated ECM-related biological functions (GO:0085029 and GO:0022617) in chondrocytes, implying therapeutic potential for OA (Fig. 3E). Furthermore, enrichment in ROS biosynthesis and metabolism (GO:0072593 and GO:1903409), together with regulation of inflammatory response and apoptotic signaling pathway (GO:0050727 and GO:2001233), suggested that K@BMT-L treatment was associated with modulation of ROS levels, inflammatory cytokine production, and apoptotic processes in OA chondrocytes (Fig. 3E). Meanwhile, enrichment of TGF-β signaling and chaperone-mediated autophagy (GO:0071604 and GO:0061684) indicated that K@BMT-L treatment was associated with TGF-β-related transcriptional changes and autophagy-associated processes (Fig. 3E). These transcriptomic data were generated from chondrocytes and therefore cannot define compartment-specific TGF-β activity in synovium, periarticular tissues, or subchondral bone.

The transcriptomic profiling revealed that K@BMT-L treatment was associated with multifaceted regulatory effects on chondrocytes. The enrichment of ECM-related processes highlights their potential to promote ECM synthesis, which is crucial for OA treatment. Concurrent modulation of ROS metabolism, inflammatory response, and apoptosis suggests a capacity to restore intracellular homeostasis and reduce catabolic stress. Moreover, the enrichment of TGF-β signaling and autophagy-related processes suggested that K@BMT-L treatment may affect protective and reparative programs in chondrocytes. However, because TGF-β signaling can also participate in synovial fibrosis, periarticular fibrosis, osteophyte formation, and subchondral remodeling depending on joint compartment and activation context, this enrichment should be interpreted as pathway association rather than definitive evidence of uniformly beneficial TGF-β activation. These findings collectively support that K@BMT-L treatment is associated with multi-target regulatory changes, providing a transcriptomic basis for its therapeutic potential in OA management while not defining the specific contribution of laser-derived heating or the compartment-specific consequences of TGF-β signaling.

### 3.4 Restoration of ECM metabolic balance by K@BMT core

**Fig. 4:**
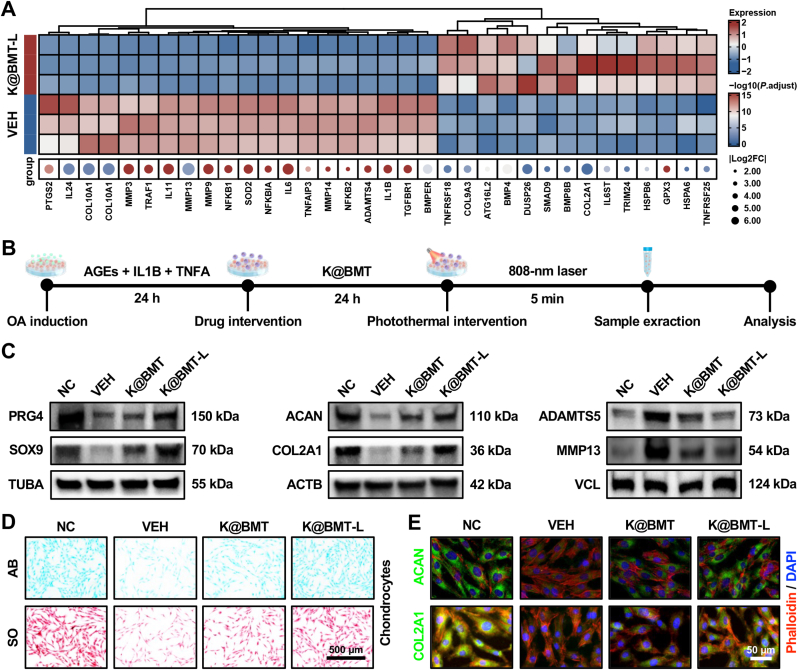
Restoration of ECM metabolic balance by K@BMT core under 808-nm laser in chondrocytes and cartilages. (A) Heatmap of representative key genes in chondrocytes. (B) Experimental schedule of OA induction, drug intervention, laser-assisted intervention, and analysis. (C) Protein levels of PRG4, SOX9, ACAN, COL2A1, ADAMTS5, and MMP13 in chondrocytes. (D) AB and SO staining images of chondrocytes for GAG and PG. (E) IF staining images of chondrocytes for ACAN and COL2A1. All data are presented as the mean ± SD (n = 3). VEH: OA-induced group treated with PBS; K@BMT-L: OA-induced group treated with K@BMT core under 808-nm laser.

According to the regulatory effects of the K@BMT core, a subset of representative key genes linked to the biological functions mentioned above was selected from the 1224 DEGs, and subsequently illustrated in a heatmap (such as *COL2A1*, *ADAMTS4*, *MMP3*, *MMP13*, *IL1B*, *IL6*, *PTGS2*, *TNFAIP3*, *TNFRSF18*, *SOD2*, *GPX3*, *SMAD9*, *TGFBR1*, *BMP4*, *ATG16L2*, and *HSPA6*) (Fig. 4A). In order to further substantiate the results obtained through transcriptome mRNA-Seq and bioinformatic analysis, a series of relevant experiments were subsequently conducted to strengthen the rigor and reliability of the results (Fig. 4B). As an initial validation of the regulatory effects of the K@BMT core on chondrocyte ECM-related markers, for chondrocytes, Western blot (WB) analysis demonstrated notable upregulation of PRG4 and SOX9 protein levels in the K@BMT and K@BMT-L groups (Fig. 4C). PRG4 is a lubricating glycoprotein secreted by surface-zone chondrocytes and synovial lining cells, which forms a boundary lubricant film in the joint cavity to reduce friction, wear, and abrasive damage to cartilage surfaces [39]. In cartilage injury and repair models, PRG4^+^ cells have been characterized as a progenitor or surface precursor population with regenerative potential, and they are capable of migrating into deeper cartilage zones and differentiating to contribute to tissue repair [40]. SOX9 is a key transcription factor for maintaining chondrocyte phenotype and differentiation, which directly regulates genes related to ECM assembly [41]. The expression of SOX9 is reciprocally regulated with multiple growth factors (including TGF-β, BMPs, and FGF), playing a critical role in cartilage repair and ECM synthesis [42]. The results of WB analysis also demonstrated that the expression levels of ECM assembly-related proteins (e.g., ACAN and COL2A1) were markedly upregulated following the K@BMT core intervention under 808-nm laser irradiation (Fig. 4C), whereas the levels of ECM disassembly-related proteins (e.g., ADAMTS5 and MMP13) were progressively downregulated (Fig. 4C), collectively indicating a pronounced anabolic shift with ECM synthesis predominating over degradation. The immunofluorescence (IF) analysis further confirmed the WB findings, showing similar expression patterns of ACAN and COL2A1 (Fig. 4E). Moreover, the results of AB and SO staining demonstrated that chondrocyte density was elevated and staining intensity was enhanced after the K@BMT core treatment under 808-nm laser irradiation, indicating that the K@BMT core facilitate chondrocyte proliferation and upregulate the expression of glycosaminoglycans (GAG) and proteoglycans (PG) (Fig. 4D).

The experimental validation provided strong support for the transcriptomic findings, demonstrating that K@BMT-based intervention exerted broad regulatory effects on ECM homeostasis in chondrocytes. The upregulation of PRG4 and SOX9 underscored their importance in maintaining the chondrocyte phenotype and enhancing cartilage repair capacity, while the simultaneous increase of ACAN and COL2A1 and decrease of ADAMTS5 and MMP13 indicated a favorable shift toward ECM synthesis rather than degradation. These findings were further supported by enhanced chondrocyte proliferation, GAG and PG accumulation, and reinforcement of the structural matrix. Notably, the inclusion of both K@BMT and K@BMT-L groups in these validation experiments allowed the ECM-regulatory effect of the chemical core and the laser-assisted intervention to be evaluated within the same experimental framework. The K@BMT group verified that the supramolecular core itself could regulate ECM metabolism in OA-related chondrocytes, whereas the K@BMT-L group showed a stronger ECM-protective response under the selected 808-nm laser-assisted condition. However, because laser-only, heat-only/temperature-matched, MnTBAP-free plus laser, and uptake/release-specific controls were not included, this difference should be interpreted as a laser-assisted enhancement rather than definitive evidence of a specific photothermal mechanism. Taken together, these findings suggest that K@BMT-based intervention, particularly under the tested laser-assisted condition, supports ECM homeostasis by regulating anabolic, catabolic, and protective processes, thereby providing experimental confirmation of its therapeutic potential in OA management.

### 3.5 Modulation of cellular homeostasis by K@BMT core

**Fig. 5:**
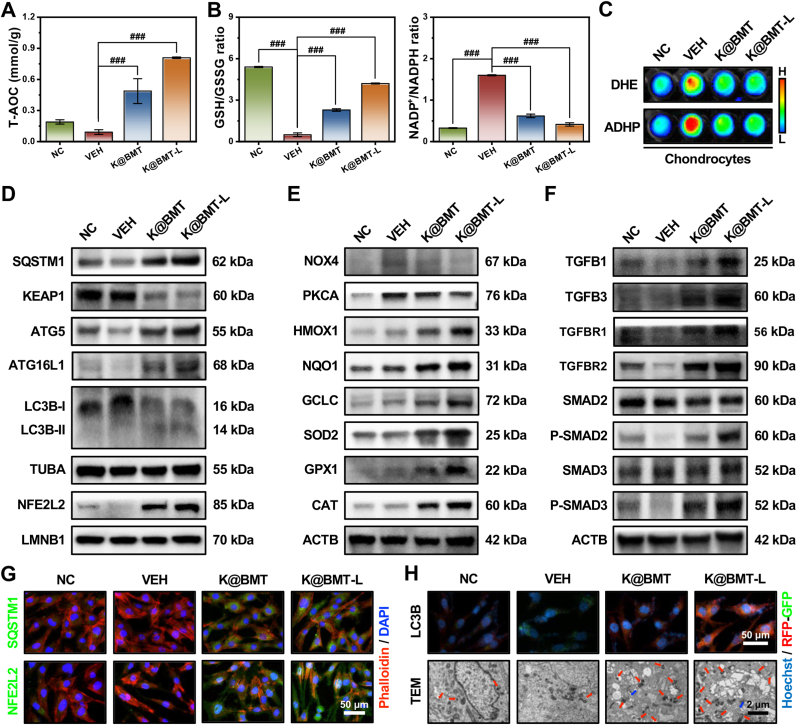
Regulation of biological functions by K@BMT core under 808-nm laser in chondrocytes. **(A)** ABTS assay of chondrocytes for T-AOC. **(B)** GSH/GSSG and NADP^+^/NADPH ratios of chondrocytes. **(C)** FI images for O_2_•^-^ and H_2_O_2_ with DHE and ADHP probes. **(D)** Protein levels of SQSTM1, KEAP1, ATG5, ATG16L1, LC3B-I/II, and NFE2L2 in chondrocytes. **(E)** Protein levels of NOX4, PKCA, HMOX1, NQO1, GCLC, SOD2, GPX1, and CAT in chondrocytes. **(F)** Protein levels of TGFB1, TGFB3, TGFBR1, TGFBR2, SMAD2, P-SMAD2, SMAD3, and P-SMAD3 in chondrocytes. **(G)** IF staining images of chondrocytes for SQSTM1 and NFE2L2. **(H)** RFP-GFP-LC3B images of chondrocytes for autophagy flux and TEM images of chondrocytes for autophagy function. All data are presented as the mean ± SD (n = 3, ^#^Compared with VEH group, ^###^*P* < 0.001).

Building on the observed improvement in ECM-related regulation, the effects of the K@BMT core on intracellular homeostasis were further investigated. Based on GSEA and GO analyses, K@BMT-L treatment was associated with the enrichment of biological processes related to antioxidant defense, autophagy, and TGF-β-associated signaling in chondrocytes. Because the TGF-β pathway can have context-dependent effects across cartilage, synovium, periarticular tissues, and subchondral bone, the present validation was focused on chondrocyte-associated signaling markers rather than whole-joint TGF-β activity. To further substantiate these transcriptomic findings, related validation experiments were performed to assess the effects of K@BMT-based intervention on key indicators associated with oxidative stress, autophagy, and TGF-β-related signaling. The antioxidant status of chondrocytes was evaluated. In the NC group, chondrocytes maintained a relatively low level of total antioxidant capacity (T-AOC), sufficient only to counteract basal oxidative metabolism. When exposed to excessive ROS accumulation, the endogenous antioxidant defense system rapidly became exhausted, resulting in a pronounced reduction of T-AOC in the VEH group. In contrast, K@BMT-L treatment was associated with a marked restoration of the antioxidant potential of chondrocytes (Fig. 5A). To further evaluate intracellular redox balance, the GSH/GSSG and NADP+/NADPH ratios were measured. Following K@BMT-based intervention, with a more pronounced response in the K@BMT-L group, the GSH/GSSG ratio increased, whereas the NADP+/NADPH ratio decreased, indicating improved intracellular redox homeostasis (Fig. 5B). Consistently, extracellular oxidative products also declined, evidenced by significantly reduced O2•^-^ and H2O2 levels following K@BMT-based intervention, particularly in the K@BMT-L group (Fig. 5C).

Next, the autophagic process in chondrocytes was further investigated. In the NC group, only a limited number of autophagosomes and autolysosomes were detected, corresponding to the basal autophagic turnover necessary for normal cellular maintenance. By contrast, chondrocytes in the VEH group exhibited markedly reduced autophagic structures, reflecting impaired autophagy-associated homeostasis under OA-like stress. After K@BMT treatment, with a more evident response in K@BMT-L-treated cells, chondrocytes displayed increased formation of autophagosomes and autolysosomes, suggesting recovery of autophagy-associated intracellular quality control (Fig. 5H). To further characterize these pathway-associated changes, the expression of key proteins related to antioxidation and autophagy was subsequently examined. WB analysis demonstrated that K@BMT-L treatment was associated with upregulation of SQSTM1, NFE2L2, ATG5, ATG16L1, and LC3B-II, together with downregulation of KEAP1 and LC3B-I expression (Fig. 5D). Meanwhile, IF analysis corroborated the WB results, exhibiting consistent expression patterns of SQSTM1 and NFE2L2 (Fig. 5G). Considering that LC3B-II accumulation alone cannot fully distinguish enhanced autophagosome formation from impaired autophagosome degradation, tandem RFP-GFP-LC3B fluorescence imaging was further performed to evaluate autophagic flux. In the VEH group, LC3B-positive puncta, especially red-only puncta, were markedly reduced, indicating impaired autophagosome maturation and autolysosome formation under OA-like conditions. In contrast, K@BMT treatment increased LC3B-positive puncta, while K@BMT-L further enhanced both yellow puncta and red-only puncta, suggesting enhanced autophagosome maturation and restored autophagic flux in the K@BMT-L group (Fig. 5H). SQSTM1 has dual roles as a selective autophagy receptor that binds LC3B via its LIR domain and as a KEAP1-sequestering protein via its KIR domain, thereby linking autophagy regulation with KEAP1/NFE2L2-associated antioxidant defense [43]. Moreover, the results showed that the K@BMT core increased the expression levels of antioxidant enzymes such as HMOX1, NQO1, GCLC, SOD2, GPX1, and CAT (Fig. 5E). This upregulation aligns with activation of the NFE2L2-ARE axis, where NFE2L2 binds antioxidant response elements (ARE) to drive transcription of antioxidant genes [44]. Meanwhile, ATG16L1 and ATG5 form part of the ATG12-ATG5-ATG16L1 complex, which catalyzes LC3B lipidation and drives autophagosome biogenesis, and their proper expression ensures effective autophagic flux and SQSTM1 turnover [45]. In addition, NOX4 and PKC expression were markedly reduced following K@BMT core intervention (Fig. 5E), indicating attenuation of pro-oxidant signaling and further supporting the restoration of redox-associated cellular homeostasis [46,47].

Subsequently, the effect of the K@BMT core on TGF-β-related signaling in chondrocytes was examined. The K@BMT-L group displayed upregulation of TGFB1 and TGFB3, along with enhanced phosphorylation of SMAD2/3 (Fig. 5F), suggesting engagement of the TGFB-SMAD2/3 axis, which may contribute to anabolic responses in chondrocytes under the current *in vitro* conditions [48,49]. However, TGF-β signaling is highly context-dependent, and pathway activation in chondrocytes does not necessarily indicate beneficial effects in all joint compartments. Inflammatory cytokines and genetic alterations have been shown to attenuate TGF-β signaling by downregulating its receptors, as evidenced in osteoarthritic cartilage [7,50]. Notably, in this study, WB analysis revealed that the K@BMT core upregulated both TGFBR1 and TGFBR2 expression (Fig. 5F), thereby supporting the involvement of TGF-β-related signaling in K@BMT-mediated chondrocyte regulation. Nevertheless, synovial fibrosis, periarticular fibrosis, compartment-specific TGF-β activation, and cartilage-specific versus synovial or subchondral effects were not evaluated; therefore, the TGF-β-related interpretation should remain restricted to chondrocyte-associated signaling changes observed in this experimental setting.

These findings were consistent with the restoration of disrupted intracellular equilibrium in osteoarthritic chondrocytes after K@BMT-based intervention. K@BMT-L treatment was associated with reactivation of the NFE2L2-ARE axis, relief of oxidative stress, and stabilization of redox homeostasis, thereby supporting mitochondrial protection and cellular metabolism. In parallel, K@BMT-L treatment was associated with upregulation of ATG5 and ATG16L1, as well as conversion of LC3B-I to LC3B-II, reflecting enhanced autophagic flux that may facilitate organelle turnover and limit ROS accumulation. Furthermore, changes in TGF-β/SMAD-related markers suggested altered growth-factor responsiveness in chondrocytes. Together, these antioxidant, autophagy-associated, and anabolic responses indicate that K@BMT-mediated protection involves multiple OA-related regulatory modules, with the potential to support chondrocyte homeostasis and cartilage matrix preservation. However, because this study did not assess synovial fibrosis, periarticular fibrosis, compartment-specific TGF-β activation, or cartilage-specific versus synovial/subchondral responses, the observed TGF-β/SMAD changes should not be generalized as uniformly beneficial joint-wide TGF-β activation. Collectively, the parallel changes in NFE2L2-ARE activity, autophagic flux, and TGF-β/SMAD responsiveness provide multi-dimensional evidence for chondrocyte-associated homeostatic remodeling after K@BMT-based intervention. This interpretation is also consistent with the multi-component design of K@BMT, which is intended to correct multiple OA-related pathological disturbances while requiring further whole-joint validation of TGF-β-related safety and compartmental effects.

### 3.6 Elimination of cellular instability by K@BMT core

Oxidative stress, inflammation, and apoptosis are closely associated pathological processes that contribute to OA progression. Given that enhancement of antioxidant defense and autophagic activity represents an important strategy to counteract these detrimental events, restoration of cellular homeostasis by the K@BMT core may help attenuate OA-related cellular instability. To further validate this hypothesis, the subsequent investigation focused on evaluating the regulatory effects of the K@BMT core on inflammatory response and apoptotic process in chondrocytes.

**Fig. 6:**
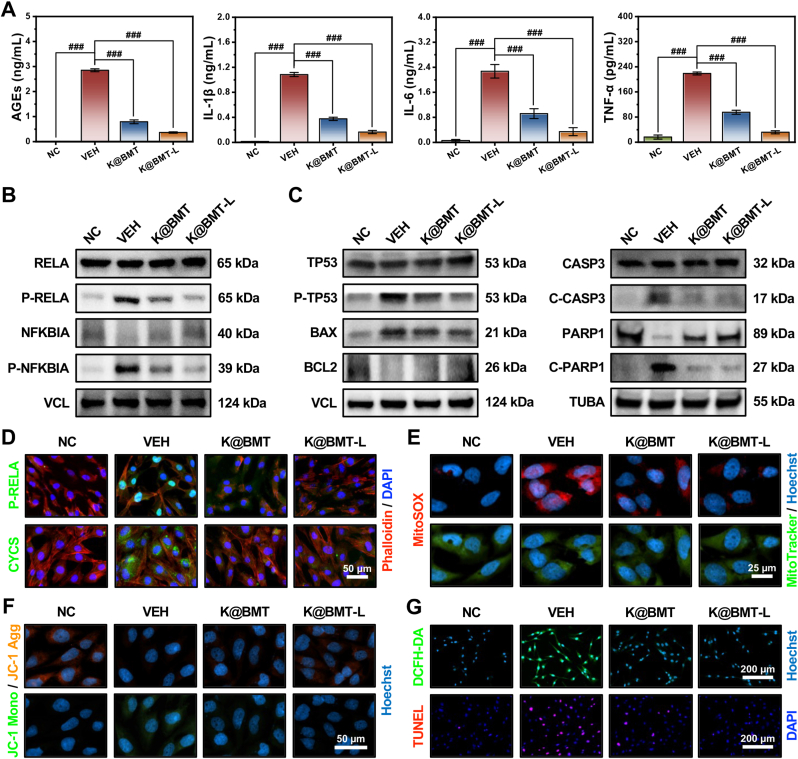
Elimination of stimulated state by K@BMT core under 808-nm laser in chondrocytes. **(A)** ELISA of chondrocytes for AGEs, IL-1β, IL-6, and TNF-α. **(B)** Protein levels of RELA, P-RELA, NFKBIA, and P-NFKBIA in chondrocytes. **(C)** Protein levels of TP53, P-TP53, BAX, BCL2, CASP3, C-CASP3, PARP1, and C-PARP1 in chondrocytes. **(D)** IF staining images of chondrocytes for P-RELA and CYCS. **(E)** MitoSOX Red staining images of chondrocytes for mitochondrial O_2_•^-^. **(F)** JC-1 staining images of chondrocytes for ΔΨm. (G) DCFH-DA staining images of chondrocytes for total ROS and TUNEL staining images of chondrocytes for apoptotic DNA fragmentation. All data are presented as the mean ± SD (n = 3, ^#^Compared with VEH group, ^###^*P* < 0.001). (For interpretation of the references to colour in this figure legend, the reader is referred to the Web version of this article.)

As the upstream driver of cellular instability, oxidative stress was first assessed at both mitochondrial and intracellular levels. MitoSOX Red staining revealed that mitochondrial O2•^-^ accumulation was markedly enhanced in the VEH group, whereas K@BMT-based intervention, particularly in the K@BMT-L group, substantially reduced mitochondrial O2•^-^ production (Fig. 6E). Consistently, DCFH-DA staining showed intense intracellular ROS fluorescence in OA-like chondrocytes, while K@BMT and K@BMT-L interventions markedly weakened the fluorescence intensity, indicating effective suppression of intracellular ROS accumulation (Fig. 6G). Since excessive ROS generation is closely associated with mitochondrial dysfunction, the mitochondrial membrane potential (ΔΨm) of chondrocytes was further assessed using the JC-1 probe. As shown in the figure, the VEH group exhibited an enhanced JC-1 monomer signal and a weakened aggregate signal, reflecting mitochondrial depolarization. In contrast, both the K@BMT and K@BMT-L groups restored the JC-1 aggregate signal and reduced the monomer signal, suggesting improved mitochondrial membrane integrity and functional stability (Fig. 6F).

Regarding inflammatory cytokines and metabolic products, enzyme-linked immunosorbent assay (ELISA) results revealed that K@BMT-L treatment markedly reduced the concentrations of Interleukin-1 beta (IL-1β), Interleukin-6 (IL-6), and tumor necrosis factor-alpha (TNF-α) in the chondrocyte culture supernatant, along with a significant decrease in the generation of AGEs (Fig. 6A). Since NF-κB signaling serves as a central mediator of inflammatory cytokine expression, related detection was performed to elucidate the involvement of this pathway in the anti-inflammatory action of the K@BMT core. WB analysis revealed a pronounced reduction in the phosphorylation levels of RELA and NFKBIA following K@BMT intervention, with a more evident response in the K@BMT-L group (Fig. 6B). This inhibitory effect was further supported by IF staining, which showed markedly suppressed nuclear translocation of P-RELA in the K@BMT and K@BMT-L groups compared with the VEH group (Fig. 6D), indicating effective attenuation of NF-κB activation.

In terms of chondrocyte apoptosis, mitochondrial damage and TP53-associated apoptotic signaling were further examined. The phosphorylation level of TP53 was significantly decreased after K@BMT intervention, accompanied by reduced expression of the pro-apoptotic protein BAX and elevated expression of the anti-apoptotic protein BCL2 (Fig. 6C). Meanwhile, the expression of cytochrome *c* (CYCS), which reflects mitochondrial apoptotic activation, was substantially downregulated in the K@BMT-L group (Fig. 6D). In parallel, the cleavage modification of CASP3 and PARP1 was markedly attenuated, suggesting that K@BMT-L treatment was associated with mitigation of apoptosis and suppression of mitochondrial-dependent caspase activation (Fig. 6C). TUNEL staining further verified this anti-apoptotic effect, as extensive apoptotic DNA fragmentation was observed in the VEH group, whereas TUNEL-positive signals were markedly reduced following K@BMT and K@BMT-L treatment (Fig. 6G).

Accumulating evidence indicates that antioxidant defense, autophagy, inflammation, and apoptosis are closely connected cellular events in OA chondrocytes. Activation of the NRF2-ARE axis promotes antioxidant gene expression and antagonizes NF-κB and p53 signaling pathways, thereby reducing inflammatory cytokines and apoptotic mediators [51]. In addition, upregulated autophagic flux helps maintain cellular homeostasis by removing damaged organelles [52]. In this study, K@BMT-L treatment was associated with restoration of disrupted intracellular homeostasis in OA chondrocytes, as reflected by enhanced antioxidant defense and autophagic activity. The observed responses included reactivation of the NRF2-ARE axis, upregulation of antioxidant genes, alleviation of oxidative stress, and stabilization of redox homeostasis, thereby supporting mitochondrial function, cellular metabolism, and reduced ROS-mediated injury. In parallel, K@BMT-L treatment was associated with upregulation of ATG5 and ATG16L1 and conversion of LC3B-I to LC3B-II, thereby indicating enhanced autophagic flux and improved clearance capacity for damaged organelles. Together with TGF-β-associated signaling changes observed in chondrocytes, these regulatory effects on antioxidant defense and autophagic flux may help attenuate oxidative stress, inflammatory responses, and apoptosis, thereby favoring ECM anabolic balance in the examined cellular context. These multi-dimensional regulatory effects indicated that K@BMT-L mitigated OA-related cellular instability by improving redox control, intracellular quality control, and stress resistance. Through this integrated regulation, the K@BMT core helped re-establish chondrocyte homeostasis and provided a favorable cellular basis for attenuating OA progression and preserving cartilage matrix integrity, while the possible fibrotic or compartment-specific consequences of TGF-β modulation remain to be clarified in future studies.

### 3.7 Design and evaluation of HM-SCHW carrier

Although the K@BMT core exhibited remarkable multi-pathway regulation and potent pharmacological efficacy in chondrocytes, its therapeutic application remains limited by rapid clearance and insufficient intra-articular (IA) retention. Given that IA injection is the primary clinical route for osteoarthritis treatment, an appropriate joint delivery carrier is crucial to improve local localization, controlled release behavior, and lubricating assistance of the K@BMT core within the cartilage region. To address this challenge, a HM carrier was designed in this study as a biocompatible carrier to encapsulate the K@BMT core, thereby constructing a novel nanomedicine, K@BMT@HM. On the one hand, SA and CS were selected as the principal components of the carrier based on their complementary physicochemical properties. SA forms ionic crosslinking networks through coordination between its carboxylate groups and multivalent cations, thereby establishing a hydrogel network that contributes to controlled molecular diffusion behavior [53,54]. In combination with CS, the formulation remains in a flowable state at room temperature, but undergoes sol-gel transition at physiological temperature, enabling *in situ* solidification after intra-articular administration [55,56]. Moreover, laser-induced local heating may further accelerate the gelation process, allowing rapid depot formation in the joint cavity. On the other hand, HA and WYRGRL were modified onto the HM carrier as auxiliary components, owing to their potential contributions to cartilage lubrication and cartilage-associated localization/retention. HA associates with phosphatidylcholine lipids at the cartilage surface to form a hydrated boundary layer, thus enhancing lubrication by reducing friction via its highly hydrated polysaccharide chains [[57], [58], [59]]. WYRGRL has been reported to recognize the alpha 1 chain of type II collagen [[60], [61], [62]], which may contribute to cartilage association. However, in the absence of a non-WYRGRL carrier, scrambled peptide control, ex vivo cartilage-binding assay, time-course retention imaging, and quantitative region-of-interest (ROI) analysis, the present data cannot distinguish WYRGRL-specific binding from hydrogel retention, particle size, viscosity, thermosensitive gelation, HA-mediated interactions, or nonspecific surface adherence. Accordingly, the carrier-related claim in this study is limited to cartilage-associated localization/retention rather than WYRGRL-mediated targeting, while sustained-release claims are limited to *in vitro* release behavior.

**Fig. 7:**
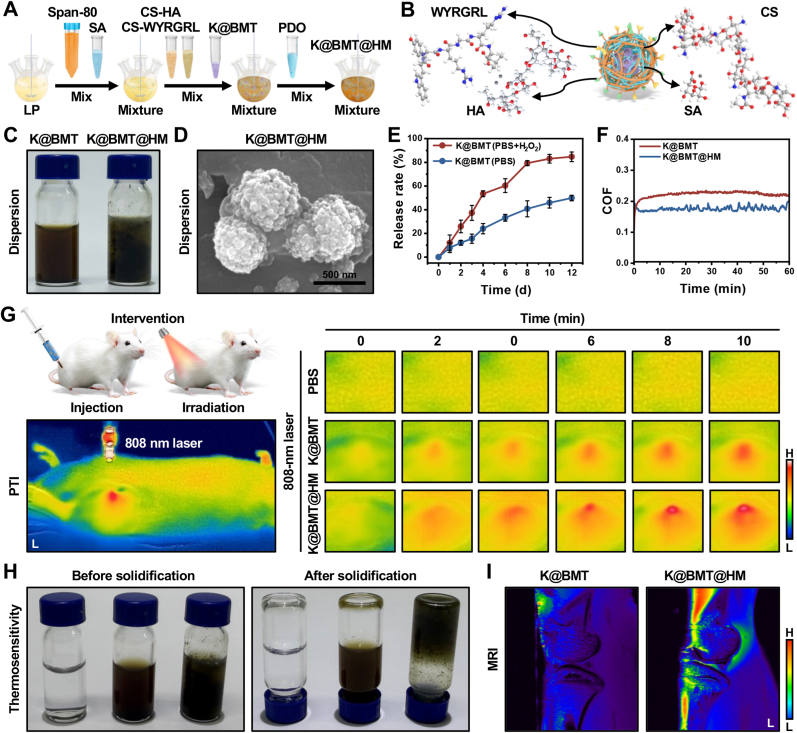
Synthesis, characterization, and performance validation of K@BMT@HM. (A) Assembly process illustration of K@BMT@HM. (B) Chemical structures of WYRGRL, HA, CS, and SA monomers and hierarchical organization of K@BMT@HM. (C) Dispersion of K@BMT and K@BMT@HM. (D) SEM images of K@BMT@HM. (E) K@BMT release rate from K@BMT@HM in PBS with or without H2O2. (F) COF of K@BMT@HM. (G) Drug and laser-assisted intervention, as well as PTI images of PBS, K@BMT, and K@BMT@HM under 808-nm laser for 10 min. (H) Panoramic views of K@BMT@HM for thermosensitivity. (I) MRI images of rat knee joints for cartilage-associated localization/retention of K@BMT@HM. All data are presented as the mean ± SD (n = 3).

The schematic illustration depicts the fabrication process of the K@BMT@HM (Fig. 7A) and the chemical structure and hierarchical organization of HA, WYRGRL, CS, and SA that form the HM carrier (Fig. 7B, Fig. S1B). The dispersion of the K@BMT@HM was subsequently examined and compared with that of the K@BMT core (Fig. 7C), and SEM revealed distinct surface architectures of K@BMT@HM (Fig. 7D). Before evaluating the thermosensitivity of the HM-SCHW carrier, the laser-responsive thermal performance of the integrated K@BMT@HM was reassessed after IA injection under 808-nm laser irradiation. Based on the concentration- and power-dependent thermal profiles established for the K@BMT core in the preceding characterization, 125 μg/mL K@BMT combined with 808-nm laser irradiation at a power density of 2 W/cm^2^ was selected as the representative condition for *in vivo* laser-assisted heating evaluation. This parameter was chosen to achieve controllable local thermal activation while avoiding excessive temperature elevation. PTI analysis showed that both K@BMT and K@BMT@HM generated localized temperature elevation in the rat knee region under 808-nm laser irradiation, while K@BMT@HM produced a more pronounced and sustained thermal response (Fig. 7G). These findings indicated that HM-SCHW integration retained the laser-responsive thermal behavior of the K@BMT core and supported local heating after intra-articular delivery. Nevertheless, these measurements mainly characterize thermal behavior and do not by themselves distinguish specific photothermal activation from nonspecific temperature elevation. To determine injectability and solidification, the thermoresponsivity of the K@BMT@HM was assessed by an inverted-vial test. Before heating, all samples were fluid dispersions. After warming, only the K@BMT@HM underwent gelation and retained its shape in the inverted-vial test, confirming the thermosensitive solidification of the HM carrier (Fig. 7H). To verify the contribution of HA to the lubricating performance of the K@BMT@HM, a tribological test was performed. The results showed that the HM-SCHW carrier maintained a relatively low and stable friction coefficient throughout the 1-h test period, whereas the HM-SCW carrier exhibited a higher friction coefficient after an initial increase and then remained at a relatively stable plateau (Fig. 7F). This comparison indicated that HA incorporation effectively reduced the friction coefficient of the HM-SCHW carrier, supporting its improved lubrication performance. Then, T1-weighted MRI was performed by taking advantage of the imaging capability of MnTBAP to evaluate the cartilage-associated localization/retention behavior of K@BMT@HM. Compared with free K@BMT, K@BMT@HM exhibited a more localized MRI signal along the articular cartilage surface of the knee joint, indicating enhanced cartilage-associated retention after integration with the HM-SCHW carrier (Fig. 7I). Nevertheless, this MRI comparison does not prove WYRGRL-specific targeting, because the observed difference may also arise from hydrogel retention, particle size, viscosity, thermosensitive gelation, HA-mediated interactions, or nonspecific surface adherence. In addition to these physicochemical characterizations, the release behavior of the K@BMT core from the HM-SCHW carrier was evaluated under physiological and oxidative stress conditions. K@BMT release was markedly accelerated in the presence of ROS, whereas release under physiological conditions remained moderate, confirming an *in vitro* sustained/ROS-responsive release profile of the HM-SCHW carrier (Fig. 7E). This *in vitro* property is relevant to the ROS-enriched OA microenvironment; however, it does not establish *in vivo* release duration or retention kinetics under continuous joint motion.

The design of the HM-SCHW carrier addresses several delivery-related constraints for IA therapy at the material-characterization level. However, the available carrier characterization does not include comprehensive rheological or mechanical testing and therefore does not substantiate a mechanical-robustness claim. SEM observations confirmed successful encapsulation of the K@BMT core, while PTI confirmed that the laser-responsive thermal behavior of the K@BMT core was retained after integration with the HM-SCHW carrier. The inverted-vial assay demonstrated a thermoresponsive sol-gel transition, indicating qualitative flow-to-gel behavior and potential *in situ* depot formation upon warming. *In vitro* release analysis further showed that the HM-SCHW carrier restrained rapid K@BMT diffusion under physiological conditions while accelerating release under ROS stimulation, supporting *in vitro* sustained and microenvironment-responsive release. Functionally, tribological testing indicated that HA incorporation stabilized the friction coefficient over 1 h, consistent with sustained boundary lubrication. MRI imaging further supported cartilage-associated localization/retention of K@BMT@HM, showing preferential accumulation of carrier-integrated K@BMT around the articular cartilage surface. However, because non-WYRGRL, scrambled peptide, ex vivo cartilage-binding, time-course retention imaging, and quantitative ROI analyses were not performed, these MRI data should not be interpreted as evidence of WYRGRL-mediated targeting. Taken together, these data indicate that the HM-SCHW carrier confers complementary attributes-thermosensitive depot formation, retained laser-responsive thermal behavior, *in vitro* sustained/ROS-responsive release, lubrication, and cartilage-associated localization/retention-thereby supporting localized delivery potential of the K@BMT core. However, because single-injection retention kinetics, reduced-frequency dosing experiments, *in vivo* release/retention time-course data, and joint-motion-mimetic release testing were not performed, the present data should not be interpreted as evidence of clinically meaningful sustained intra-articular delivery. Therefore, the carrier evaluation supports localized delivery, cartilage-associated localization/retention, and *in vitro* sustained/ROS-responsive release, whereas WYRGRL-specific targeting and *in vivo* durability of intra-articular retention require further validation. In addition, because rheology, storage/loss modulus, gelation temperature/time, injectability force, swelling ratio, degradation kinetics, compressive/shear stability, enzymatic degradation, and release under mechanical loading were not evaluated, the present data support only the tested thermosensitive behavior, lubrication, cartilage-associated localization/retention, and *in vitro* sustained/ROS-responsive release, rather than mechanical robustness of the HM-SCHW carrier.

### 3.8 Attenuation of OA process by K@BMT@HM nanomedicine

**Fig. 8:**
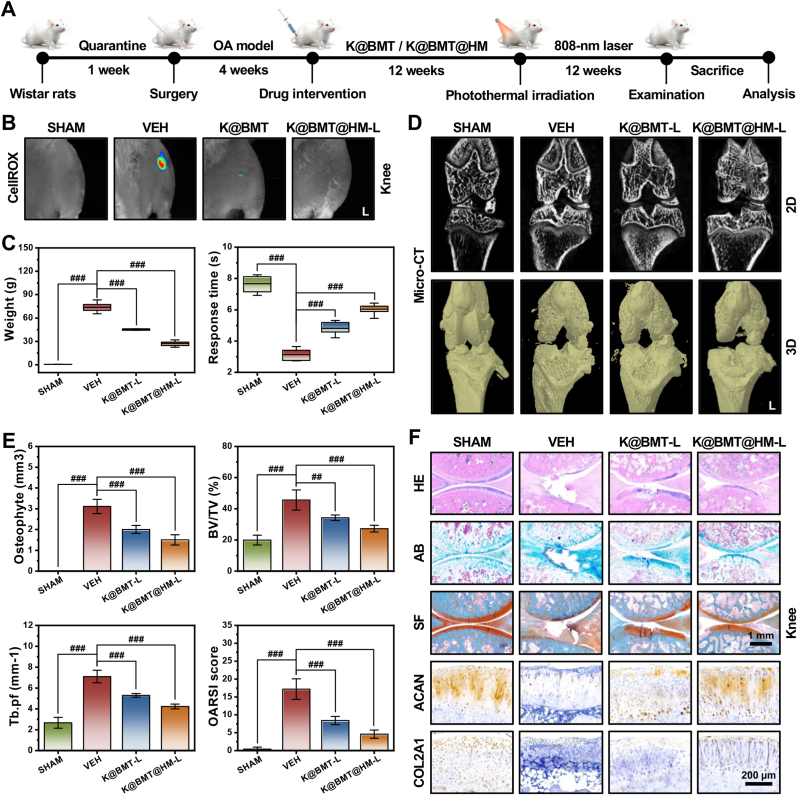
Protection of ECM and improvement of knee function by K@BMT@HM under 808 nm laser in rat knees (A) Experimental schedule of establishment of OA rat models, drug intervention, laser-assisted intervention, knee examination, and analysis. (B) FI images of in rat knee joints for ROS with CellROX probes. (C) Difference between the weight placed on the two hindlimbs determined by the weight-bearing test, and pain response time of rat left hindlimbs determined by the hot-plate test. (D) Micro-CT images of rat knee joints for 2D fault and 3D reconstruction. (E) Total osteophytes volume, BV/TV, Tb.pf, and OARSI score of rat knee joints. (F) HE, AB and SF staining images of rat knee joints for cartilage degeneration degree of cartilage and expression level of GAG and PG, as well as IHC staining images of rat knee joints for ACAN and COL2A1. All data are presented as the mean ± SD (n = 5, #Compared with VEH group, ##P < 0.01, ###P < 0.001).

The preceding cellular experiments demonstrated that K@BMT-based interventions regulated multiple OA-related pathological events in chondrocytes, including inflammation, oxidative stress, apoptosis, and ECM metabolic imbalance. However, the complex articular environment, including rapid synovial fluid clearance, enzymatic degradation, and limited cartilage penetration, may restrict the therapeutic performance of IA administered nanomedicines. Therefore, after the cellular validation experiments had evaluated the chemical core and the laser-assisted intervention using K@BMT and K@BMT-L groups, the *in vivo* study was further designed to examine the carrier-dependent therapeutic performance of the integrated K@BMT@HM system. Specifically, K@BMT-L and K@BMT@HM-L were compared in the rat OA model as a small-animal proof-of-concept setting to determine whether the HM-SCHW carrier could improve the *in vivo* performance of K@BMT within the selected 808-nm laser-assisted regimen. Because the *in vivo* design did not include K@BMT@HM without laser, laser-only, heat-only/temperature-matched, or MnTBAP-free plus laser controls, this comparison was intended to evaluate the integrated system under the tested treatment condition rather than to isolate the independent contribution of photothermal activation. In addition, the therapeutic study used repeated intra-articular administration twice per week for 12 weeks; therefore, it should be considered a repeated-treatment efficacy regimen rather than direct evidence of single-injection persistence or clinically meaningful sustained intra-articular delivery. The HM-SCHW carrier, featuring thermosensitive solidification, *in vitro* sustained/ROS-responsive release, lubrication, and cartilage-associated localization/retention potential, was expected to support local exposure under the tested dosing schedule. Subsequently, the therapeutic efficacy and biosafety of K@BMT@HM-L were evaluated in OA rat models (Fig. 8A). Biosafety of K@BMT and K@BMT@HM was comprehensively assessed through blood tests and histological examination. Blood analyses confirmed that both the K@BMT-L and K@BMT@HM-L groups exhibited normal hematological parameters, including WBC, RBC, Hb, Hct, MCH, and PLT (Fig. S3A), as well as serum biochemical markers for liver (ALT, AST, ALP) and kidney (CREA, UREA) function and serum Mn levels (Fig. S3B), all comparable to SHAM and VEH groups. Furthermore, HE staining revealed no apparent histopathological abnormalities in major organs following intra-articular injection of K@BMT@HM, demonstrating that the treatment is well tolerated and does not induce organ toxicity (Fig. S4A). Taken together, these endpoint assessments indicated that repeated IA delivery of K@BMT and K@BMT@HM was well tolerated during the 12-week observation period, with no detectable hematological abnormality, liver/kidney dysfunction, serum Mn elevation, or major organ toxicity. These findings provided supportive systemic safety evidence for the therapeutic evaluation of K@BMT@HM *in vivo*. This experimental design was not intended to reproduce the optical and anatomical barriers of human OA joints, and therefore cannot determine whether 808-nm laser irradiation would reach human articular cartilage at therapeutically relevant and safe thermal levels. Importantly, the *in vivo* comparison was designed to evaluate the integrated K@BMT@HM system under the tested regimen, rather than to assign component-specific efficacy to BDMC, MnTBAP, KRFK, 808-nm laser activation, CS/SA hydrogel formation, HA-mediated lubrication, or WYRGRL-associated localization.

With systemic and organ-level safety established, the therapeutic efficacy of the K@BMT@HM nanomedicine was subsequently evaluated in OA rat models to determine its ability to mitigate oxidative stress, restore joint function, and protect cartilage integrity. According to the *in vivo* FI result, the level of ROS in rat knee joints was reduced in the K@BMT-L and K@BMT@HM-L groups (Fig. 8B). The weight-bearing test revealed a markedly greater weight bearing difference between the hind limbs in the VEH group compared with the SHAM group, whereas K@BMT-L and K@BMT@HM-L treatment improved bilateral balance (Fig. 8C). The hot plate test showed that rats in the VEH group exhibited a markedly shorter latency to pain response compared with the SHAM group, whereas the K@BMT-L or K@BMT@HM-L groups notably prolonged the reaction time, indicating improved analgesic and protective effects (Fig. 8C). In addition, the right knee joints of rats were examined by micro-computed tomography (micro-CT), including 2D scanning and 3D reconstruction (Fig. 8D). From the macroscopic perspective, micro-CT imaging revealed a pronounced increase in osteophyte volume in the VEH group, whereas the K@BMT-L core or K@BMT@HM-L group exhibited a substantial reduction in osteophyte formation (Fig. 8E). Meanwhile, the bone trabecular analysis showed consistent trends with the imaging results (Fig. 8E). Besides, the degeneration of cartilage is also evaluated through Osteoarthritis Research Society International (OARSI) score, and the result indicated that cartilage degeneration was markedly alleviated following treatment with the K@BMT core or K@BMT@HM under 808-nm laser, suggesting that K@BMT-L and K@BMT@HM-L treatment was associated with protection against cartilage structural degradation (Fig. 8E). From the microscopic perspective, pathological analysis revealed the degradation of GAG and PG in the knee joints of the VEH group, whereas this degradation was markedly attenuated in the K@BMT-L or K@BMT@HM-L group (Fig. 8F). Moreover, the proportions of ACAN+ and COL2A1+ chondrocytes were substantially elevated in the cartilage of rats treated with K@BMT-L or K@BMT@HM-L, whereas the proportions of MMP13+ chondrocytes were reduced (Fig. 8E, Fig. S4B).

Notably, under the tested 808-nm laser-assisted condition, both K@BMT-L and K@BMT@HM-L exhibited therapeutic effects *in vivo*, whereas K@BMT@HM-L showed more pronounced cartilage-protective efficacy. This improvement is most conservatively interpreted as a carrier-dependent enhancement within the laser-assisted repeated-treatment regimen, rather than definitive proof that specific photothermal activation, clinically durable sustained delivery, or WYRGRL-mediated targeting is the sole or dominant mechanism. In the absence of laser-only, heat-only/temperature-matched, MnTBAP-free plus laser, K@BMT@HM without laser, and K@BMT@HM plus laser versus K@BMT@HM without laser comparisons, the effects of laser irradiation cannot be fully separated from nonspecific mild hyperthermia, altered release kinetics, enhanced cellular uptake, heat-shock protein-mediated stress responses, or other laser-associated influences. Similarly, because the *in vivo* study used high-frequency intra-articular injections twice weekly for 12 weeks and did not include single-injection retention kinetics, reduced-frequency dosing comparisons, *in vivo* release/retention time-course imaging, or joint-motion-mimetic release testing, the current results cannot substantiate a clinically meaningful sustained intra-articular delivery claim. In addition, the MRI-based localization results should be interpreted as cartilage-associated localization/retention, not as direct evidence of WYRGRL-specific targeting, because non-WYRGRL, scrambled peptide, ex vivo cartilage-binding, time-course retention, and quantitative ROI controls were not included. The HM-SCHW carrier may improve local exposure through thermosensitive depot formation, lubrication, and cartilage-associated localization/retention, but the duration of *in vivo* retention, release, and the specific contribution of WYRGRL remain to be defined. Further optimization of the dosing interval, carrier rheological and mechanical characterization (including storage/loss modulus, gelation temperature/time, injectability force, swelling ratio, degradation kinetics, compressive/shear stability, enzymatic degradation, and release under mechanical loading), temperature-matched controls, component-deletion controls, release/uptake tracing, single-injection retention studies, reduced-frequency dosing experiments, joint-motion-mimetic release assays, larger joint models, ex vivo thick-tissue penetration experiments, optical-thermal simulations, non-WYRGRL or scrambled peptide controls, ex vivo cartilage-binding assays, time-course retention imaging, and quantitative ROI analysis will help clarify delivery durability, cartilage association, and the mechanism of laser assistance. Collectively, these findings indicate that K@BMT@HM-L improves cartilage integrity and joint function *in vivo* under the tested repeated-treatment conditions, while sustained delivery should be concluded only from the *in vitro* release data and cartilage association should be described as localization/retention until stronger *in vivo* retention and targeting-specific evidence is available. Thus, the present *in vivo* data should be interpreted as small-animal proof-of-concept evidence rather than direct support for clinically sustained intra-articular delivery, WYRGRL-mediated targeting, or clinical photothermal applicability in human OA joints. Therefore, the observed *in vivo* efficacy should be regarded as the outcome of the integrated formulation and treatment protocol, while the contribution, redundancy, or indispensability of individual chemical, photothermal, hydrogel, lubrication, and cartilage-association elements remains unresolved without systematic deletion or factorial component controls.

## 4 Conclusion

In summary, this study developed a multifunctional nanomedicine that integrates a multi-component bioactive core with a multifunctional delivery carrier for OA intervention. The K@BMT core, composed of KRFK, BDMC, and MT, regulated multiple OA-related cellular events, as reflected by enhanced antioxidant defense, improved autophagic flux, and modulation of TGF-β-associated signaling, thereby mitigating oxidative stress, inflammatory responses, and apoptosis while favoring ECM anabolic balance in the examined chondrocyte-centered models. However, because TGF-β signaling is context-dependent and may exert divergent effects in cartilage, synovium, periarticular tissues, and subchondral bone, the present findings should not be interpreted as demonstrating uniformly beneficial joint-wide TGF-β activation. The HM-SCHW carrier, constructed from CS, SA, HA, and WYRGRL, provided thermosensitive depot formation, lubrication, cartilage-associated localization/retention, and *in vitro* sustained/ROS-responsive release, thereby supporting localized delivery potential of the K@BMT core. The integrated K@BMT@HM nanomedicine demonstrated carrier-dependent therapeutic advantages, improving chondrocyte homeostasis, attenuating OA-associated cartilage deterioration, and improving joint function *in vivo* under a repeated-treatment regimen. Collectively, these findings suggest that K@BMT@HM may serve as a preclinical proof-of-concept platform for multimodal OA intervention. Importantly, the present data should be interpreted as demonstrating laser-assisted enhanced effects under the tested conditions rather than proving a specific photothermal mechanism. Future studies incorporating laser-only, heat-only/temperature-matched, MnTBAP-free plus laser, K@BMT@HM without laser, K@BMT@HM plus laser versus K@BMT@HM without laser, release/uptake tracing, and heat-shock/stress-response pathway controls are required to distinguish photothermal activation from nonspecific hyperthermia, enhanced release or uptake, and stress-response effects. Moreover, because the *in vivo* study used intra-articular injections twice per week for 12 weeks and did not include single-injection retention kinetics, reduced-frequency dosing experiments, *in vivo* release/retention time-course data, or joint-motion-mimetic release testing, sustained-delivery conclusions should be limited to the *in vitro* sustained/ROS-responsive release results. Likewise, because rheology, storage/loss modulus, gelation temperature/time, injectability force, swelling ratio, degradation kinetics, compressive/shear stability, enzymatic degradation, and release under mechanical loading were not characterized, the present manuscript does not claim mechanical robustness of the HM-SCHW carrier. In addition, because the MRI study lacked a non-WYRGRL carrier, scrambled peptide control, ex vivo cartilage-binding assay, time-course retention imaging, and quantitative ROI analysis, the localization result should be described as cartilage-associated localization/retention rather than WYRGRL-mediated targeting. Finally, the photothermal component of this strategy has currently been validated only in a rat small-joint model; because human OA joints contain deeper cartilage shielded by skin, adipose tissue, synovium, and other tissues, these results cannot be directly extrapolated to human OA. Future studies should incorporate ex vivo human-relevant thick-tissue penetration assessment, large-joint models, optical-thermal simulations, targeting-specific controls, *in vivo* retention/release analyses, comprehensive carrier rheology/mechanical characterization, and whole-joint assessment of TGF-β-related outcomes, including synovial fibrosis, periarticular fibrosis, compartment-specific TGF-β activation, and cartilage-specific versus synovial/subchondral responses, to determine whether safe, effective, and clinically durable intra-articular delivery can be achieved in clinically relevant joints. Overall, the present work validates K@BMT@HM as an integrated proof-of-concept nanomedicine, but it does not define the quantitative contribution, necessity, or indispensability of each individual component. Future studies using single-component, two-component, deletion, and factorial comparison controls will be required to determine how BDMC, MnTBAP, KRFK, 808-nm laser activation, the CS/SA hydrogel matrix, HA, and WYRGRL each contribute to therapeutic efficacy, delivery behavior, and safety.

## CRediT authorship contribution statement

**Yibo Ma:** Conceptualization, Data curation, Methodology, Writing – original draft. **Renjie Zhang:** Conceptualization, Data curation, Validation, Writing – original draft. **Songjie Han:** Data curation, Validation, Visualization, Writing – original draft. **Ziyu Zhuang:** Data curation, Formal analysis, Validation. **Zhenggang Wang:** Investigation, Writing – original draft. **Chenyue Xu:** Data curation, Formal analysis, Visualization. **João Conde:** Conceptualization, Resources, Supervision, Writing – review & editing. **Jinyu Li:** Funding acquisition, Methodology, Supervision, Writing – review & editing. **Qiaoli Zhai:** Methodology, Resources, Supervision, Writing – review & editing. **Changjian Chen:** Conceptualization, Funding acquisition, Project administration, Writing – review & editing.

## Declaration of competing interest

The authors declare the following financial interests/personal relationships which may be considered as potential competing interests: Joao Conde reports administrative support, equipment, drugs, or supplies, and statistical analysis were provided by NOVA University Lisbon Medical School. Joao Conde reports a relationship with NOVA University Lisbon Medical School that includes: employment. João Conde is a co-founder and shareholder of TargTex S.A. - Targeted therapeutics for Glioblastoma Multiforme. João Conde is a member of the Global Burden Disease (GBD) consortium of the Institute for Health Metrics and Evaluation (IHME), University of Washington (US) and is on the Scientific Advisory board of Vector Bioscience Cambridge. The other authors declare no competing financial interest. If there are other authors, they declare that they have no known competing financial interests or personal relationships that could have appeared to influence the work reported in this paper.

## Data Availability

Data will be made available on request.
