# Supplementary material for: Multiregulatory hydrogel supramolecular nanomedicine for reprogramming cartilage homeostasis in osteoarthritis
Source: Mater Today Bio. 2026 Jun 20;39:103380. doi: 10.1016/j.mtbio.2026.103380 (PMC13316178; doi:10.1016/j.mtbio.2026.103380)
Supplement: Multimedia component 1 [file mmc1.pdf]

Supplementary Information

1. Supplementary methods

1.1. Synthesis of nanomedicine core

Bisdemethoxycurcumin (BDMC, MedChemExpress, USA) and MnTBAP (MedChemExpress, USA) were dissolved in methanol (MeOH, aladdin, China) and the stock solution with BDMC/MnTBAP molar ratios (1:1) was obtained after ultrasound assisted fusion. Subsequently, the stock solution (100 μL) was added dropwise to ultrapure water (1 mL) under magnetic stirring at room temperature, then the ultrapure water with BDMC and MnTBAP was oscillated by magnetic stirring for 1 h and ultrasonic oscillation for 3 h and the BDMC-MnTBAP (BMT) core were obtained and dried into powders. BDMC and MnTBAP were also dissolved in MeOH and the stock solution with BDMC/MnTBAP molar ratios (1:1) was obtained after ultrasound assisted fusion. Differently, the stock solution (100 μL) was added dropwise to ultrapure water (1 mL) containing KRFK under magnetic stirring at room temperature, then the ultrapure water with BDMC, MnTBAP, and KRFK was oscillated by magnetic stirring for 1 h and ultrasonic oscillation for 3 h and the KRFK@BDMC-MnTBAP (K@BMT) core were obtained and dried into powders.

1.2. Characterization of nanomedicine core

The morphology of BMT and K@BMT core suspended in water were tested via low-voltage transmission electron microscopy (LV-TEM, JEM-1200EX, JEOL, Japan), high-resolution TEM (HR-TEM), and high-angle annular dark-field scanning transmission electron microscope (HAADF-STEM, Talos F200X, FEI, USA). Energy Dispersive Spectrometer (EDS, Talos F200X, FEI, USA) was applied for elemental mappings of BMT and K@BMT core. The particle sizes and zeta potentials of BMT and K@BMT core were determined at 25°C using dynamic light scattering (DLS) with laser particle size analyzer (Mastersizer 3000, Malvern Panalytical, UK). The absorption spectrum of KRFK, BDMC, MnTBAP, BMT and K@BMT core was measured in the wavelength range of 200 - 600 nm to determine its maximum absorption wavelength with Ultraviolet-visible (UV-Vis) spectroscopy (Evolution One, Thermo Fisher Scientific, USA). The valence state of manganese element was characterized through X-ray photoelectron spectroscopy (XPS) (ESCALAB 250Xi, Thermo Scientific, USA).

1.3. Performance verification of nanomedicine core

To evaluate the stability, K@BMT cores were suspended in PBS or DMEM at 250 μg/mL. Particle

size and morphology were monitored over 7 days to assess stability. To evaluate the ROS responsiveness of the K@BMT core, dihydroethidium (DHE, Beyotime, China) was used to visualize the level of $O_2^{\bullet -}$ under the fluorescence spectral of DHE (Ex/Em= 300/610 nm), while the Amplex Red (ADHP, Beyotime, China) was used to detect the level of H_2O_2 under the fluorescence spectral of ADHP (Ex/Em= 571/585 nm) via the IVIS Spectrum (PerkinElmer, USA). For valence state characterization, XPS was performed to determine the oxidation states of manganese (Mn 2p) in K@BMT before and after ROS response. To further assess ROS-triggered release, K@BMT suspensions were incubated under the same conditions, and aliquots were collected at defined time points (0–12 h), and the cumulative release of KRFK, BDMC, and MnTBAP was quantified. To evaluate the photothermal effect of K@BMT core, irradiation was performed using an 808 nm laser at the power density of 2 W/cm^2 for a duration of 6 min across varying concentrations of the K@BMT core (0, 31.25, 62.5,125, and 250 μg/mL). The temperature of the solution was recorded at 30-s intervals using thermal camera and thermal images were captured. Additionally, in order to assess the correlation between temperature changes in the K@BMT core and laser power density, the temperature of 250 μg/mL K@BMT core was measured under different laser power densities (0.5, 1, 1.5, 2, and 2.5 W/cm^2). The temperature of solution was monitored every 30 s with thermal camera. The photothermal stability of K@BMT core was further evaluated through five repeated heating/cooling cycles.

1.4. Cell culture and treatment

The human chondrocytes (Immocell, China) were cultivated with dulbecco's modified eagle's medium (DMEM, Gibco, USA) containing 15% fetal bovine serum (FBS, Gibco, USA) and antibiotics (Beyotime, China). The chondrocytes were identified via Safranin O (SO, Solarbio, China) and alcian blue (AB, Solarbio, China) staining. In order to directly reveal the regulatory effects of K@BMT core on OA therapy, the chondrocytes were divided into 4 groups, including NC, VEH, K@BMT, and K@BMT-L group. Next, chondrocytes from VEH, K@BMT, and K@BMT-L group were induced into OA phenotype with IL1B (10 ng/mL, MedChemExpress, USA), TNFA (20 ng/mL, MedChemExpress, USA), and AGE (50 μg/mL, Bioss, China) for 24 h, then intervened simultaneously with vehicle (phosphate-buffered saline, PBS), K@BMT, and K@BMT under 808-nm laser (6 min) for 24 h.

1.5. Cell viability assay

Cell counting kit-8 (CCK-8, Dojindo, Japan) was employed to evaluate viability of chondrocytes after K@BMT intervention with or without 808-nm laser irradiation. The cells (5000 cells/well) were placed into 96-well plates (Corning, USA) and incubated for 24 h and incubated with K@BMT (50,

100, 150, 200, and 250 μg/mL) for 24 h, while half of the cells were irradiated with 808-nm laser for 6 min. Then, CCK-8 solution was added into each well and incubated for 2 h and the absorbance was read with a microplate reader (Thermo Fisher Scientific, USA) at 450 nm. The optimal concentration ranges of K@BMT intervention with or without 808 nm laser irradiation was determined through the formula: Cell viability (%) = (A_{sample} − A_{blank}) / ($A_{control}$ − A_{blank}) × 100%, where A_{sample}, A_{blank} and $A_{control}$ refer to the absorbance of test well (including K@BMT, cells and DMEM), control well (including cells and DMEM) and blank well (only including DMEM) respectively.

1.6. Transcriptome mRNA sequencing (mRNA-seq) and bioinformatics analysis

Chondrocytes from VEH and K@BMT-L groups were washed with PBS after removing the DMEM, and then total RNA were extracted with TRIzol reagent followed by the inspection of integrity and purity. The mRNA was purified from total RNA and transcribed into cDNA, then the cDNA fragments were purified with AMPure XP system (Beckman Coulter, Beverly, USA) and amplified through PCR to construct the library. The clustering of the index-coded samples was performed on a cBot Cluster Generation System using TruSeq PE Cluster Kit v3-cBot-HS (Illumia, USA) and the library preparations were sequenced on an Illumina Novaseq platform after cluster generation and 150 bp paired-end reads were generated. Differential expression analysis of VEH group and K@BMT-L group was performed using the "DESeq2" package in R 4.3.3. Genes (P < 0.05) found by DESeq2 were assigned as differentially expressed, while the expression level of genes was displayed as volcano plots and heatmaps by ggplot2. The differentially expressed genes (DEGs) were also performed for Gene Ontology (GO) and Kyoto Encyclopedia of Genes and Genomes (KEGG) enrichment analysis using "clusterProfiler" package, and all terms with P < 0.05 were screened. Gene set enrichment analysis (GSEA) software (ver. 4.10.1, Broad Institute, MIT) was applied to perform GSEA and the results of GSEA were plotted via R 4.3.3.

1.7. Western blot (WB) analysis

The chondrocytes from each group were collected, then total proteins were extracted with RIPA lysis buffer (Beyotime, China) containing protease and phosphatase inhibitors (Beyotime, China) and the concentration of proteins was determined using BCA reagent (Beyotime, China). Denatured proteins and protein ladders (Thermo Fisher Scientific, USA) were added to the precast protein gels (Yeasen, China), then connected the electrophoresis apparatus (Bio-Rad, USA) and set the voltage to 150 V. After electrophoresis, the gels were removed and transferred to the mini gel holder cassettes (Bio-Rad, USA) with the methanol-activated PVDF membranes (Millipore, USA), then assembled the transfer equipment (Bio-Rad, USA) and adjusted the current to 300 mA. After transfer, the PVDF

membranes were blocked with blocking buffer (Beyotime, China) for 15 min, then immersed in the corresponding primary antibody and incubated at 4 °C overnight. The above membranes were washed with TBST buffer 3 times on the morrow and pumped with the matching secondary antibody (Abcam, USA) and shaken at room temperature for 1 h. The PVDF membranes incorporating the secondary antibody were immersed in the enhanced chemiluminescence (ECL) solution (Beyotime, China) and placed in the imaging system (Bio-Rad, USA). The information of primary antibody applied for Western blot assay were shown in Supplementary Information (Table S1).

1.8. Immunofluorescence (IF) staining

For cell climbing tablets, 4% paraformaldehyde (Biosharp, China) was applied for fixing the cells for 20 min after removing the DMEM from 12-well plates (Corning, USA), and then Triton X-100 (Beyotime, China) was used to permeate the cell membrane and nuclear membrane. After the preparation of specimens, the cell climbing tablets were blocked with blocking buffer (Beyotime, China) for 20 min and incubated with the corresponding primary antibody at 4 °C overnight. The above climbing tablets were washed with TBST buffer 3 times the next day and pumped with the matching secondary antibody coupled FITC or CY3 (Absin, China) at room temperature for 1 h in the absence of light. Rhodamine phalloidin (Thermo Fisher Scientific, USA) was added to the cell climbing tablets bearing chondrocytes for 1 hour at room temperature and washed with TBST buffer 3 times. Next, DAPI (Thermo Fisher Scientific, USA) was added to all cell climbing tablets for 15 min at room temperature away from light. The above climbing tablets, sealed with resin, were scanned into images through research slide scanner (Olympus, Japan), while the images of each channel were merged. The information of primary antibody applied for immunofluorescence assay were shown in Supplementary Information (Table S2).

1.9 Enzyme linked immunosorbent assay (ELISA)

Chondrocyte culture supernatants were collected and centrifuged at 3000 g to remove impurities. The levels of AGEs, IL1B, IL6, and TNFA were quantified using the high sensitive ELISA kit for advanced glycation end product (Cloud-Clone Corp, China) as well as the human IL-1β, IL-6, and TNF-α ELISA kit (Beyotime, China) respectively. Absorbance was measured with a microplate reader (Thermo Fisher Scientific, USA). The level of IL1B, IL6, and TNFA was calculated according to the standard curve.

1.10. Reactive oxygen species (ROS) assay

For chondrocyte culture supernatants, DHE and ADHP probes were also employed to detect the levels of $O_2{\bullet}^-$ and H_2O_2 respectively, and the fluorescence signals were captured using an IVIS Spectrum. For chondrocytes, total intracellular ROS and mitochondrial $O_2{\bullet}^-$ levels were evaluated

using a ROS Assay Kit (Beyotime, China), MitoSOX Red (Yeasen, China), and Hoechst 33342 (Thermo Fisher Scientific, USA). Cells were incubated with the probes for 15 min, followed by three washes with serum-free DMEM. Fluorescence images were captured using a fluorescence microscope (Zeiss, Germany). DCFH-DA (Ex/Em = 488/525 nm) was used to detect total ROS, MitoSOX Red (Ex/Em = 510/580 nm) for mitochondrial superoxide, and Hoechst (Ex/Em = 350/461 nm) for nuclear staining.

1.11. Mitochondrial membrane potential (ΔΨm) assay

The chondrocytes from each group were washed with PBS buffer after removing the DMEM, then incubated with JC-1 (Beyotime, China) for 20 min and washed with DMEM 3 times. The situation of mitochondrial membrane potential within chondrocytes was observed under the fluorescence spectral of FITC and CY3 via a fluorescence microscope.

1.12. TEM Analysis

The chondrocytes after the intervention were fixed in 2.5% glutaraldehyde (pH = 7.4, Servicebio, China) for 24 h and 1% osmium tetroxide for 2 h. After dehydration, infiltration, imbedding and sectioning, the sections were stained with uranyl acetate and lead citrate, while observed using a transmission electron microscope (Hitachi, Japan).

1.13. Antioxidant assay

The total antioxidant capacity (T-AOC) of chondrocytes was determined using total antioxidant capacity assay kit (Beyotime, China) and calculated according to the standard curve. The levels of GSH and GSSG in chondrocytes were detected using GSH and GSSG assay kit (Beyotime, China) and the absorbance was read at 412 nm. The levels of $NADP^+$ and NADPH in chondrocytes were detected with $NADP^+$/NADPH assay kit (Merck, USA) and the fluorescence (Ex/Em= 530/585 nm) was determined. The GSH/GSSG ratio and $NADP^+$/NADPH ratio were calculated according to the standard curve.

1.14. TdT-mediated dUTP nick-end labeling (TUNEL) assay

Apoptotic chondrocytes were detected using a TUNEL-FITC apoptosis detection kit (Vazyme, China) following the manufacturer’s instructions. Stained sections were mounted and scanned with a digital slide scanner, and the fluorescence channels were merged for imaging.

1.15. Tandem fluorescent LC3B autophagic flux assay

For chondrocytes, autophagic flux was assessed using a tandem fluorescent LC3B reporter. Chondrocytes were transduced with Premo Autophagy Tandem Sensor RFP-GFP-LC3B Kit (Thermo Fisher Scientific, USA). Briefly, chondrocytes were incubated with the RFP-GFP-LC3B reagent in

complete culture medium and maintained overnight to allow expression of the fluorescent fusion protein. After incubation, cells were washed with serum-free DMEM to remove excess reagent. Fluorescence images were captured using a fluorescence microscope under appropriate filter settings for GFP (green) and RFP (red). Yellow puncta (GFP+RFP) and red-only puncta (RFP only) were used to distinguish autophagosomes and autolysosomes, respectively, thereby enabling evaluation of autophagic flux.

1.16. Preparation of nanomedicine

To synthesize functionalized chitosan (CS, Aladdin, China), 100 mg of hyaluronic acid (HA, MedChemExpress, USA) and WYRGRL (MedChemExpress, USA) were each dissolved in 10 mL of 2-(N-morpholino)ethanesulfonic acid (MES, Merck, USA) buffer solution. Then, 120 mg of N-hydroxysuccinimide (NHS, MedChemExpress, USA) and 100 mg of 1-ethyl-3-(3-dimethylaminopropyl)carbodiimide hydrochloride (EDC·HCl, Merck, USA) were added, and the mixture was stirred at 37 °C for 30 min to activate the carboxyl groups. Subsequently, 1 g of CS was added, and the reaction was allowed to proceed overnight at 37 °C. The resulting solution was dialyzed using a dialysis membrane for 3 days and lyophilized to obtain the HA and WYRGRL–modified CS, called CS-HA and CS-WYRGRL. The inverse emulsion–crosslinking method was employed to construct hydrogel microspheres (HM), which served as carriers to combine with the K@BMT core, ultimately forming the nanomedicine. Specifically, precisely measured 30 mL of liquid paraffin (Aladdin, China) was used as the oil phase and poured into a dry 100 mL three-neck flask. The flask was placed in a thermostatic water bath set at 45 °C and maintained at a constant temperature. A mechanical stirrer was installed in the central neck, with the stirring paddle adjusted to the center of the flask bottom. The mechanical stirrer was then started and set to 500 rpm to provide an initial mixing environment for the subsequent emulsification process. Under continuous stirring, 2 mL of Span-80 (Aladdin, China) and 2 mL of sodium alginate (SA, MedChemExpress, USA) solution (200 mg/mL) were sequentially added to the paraffin oil phase using a pipette. The mixture was stirred for 5 min to ensure thorough dispersion of the emulsifier within the oil phase, yielding a uniform emulsion system. Next, while maintaining the water bath temperature and stirring speed, the following aqueous solutions were slowly added dropwise into the oil phase using a pipette in sequence: 1 mL of CS solution (200 mg/mL), 1 mL of functionalized CS (500 mg/mL, with a mass ratio of CS-HA to CS-WYRGRL of 1:1), 1 mL of $CaCl_2$ (Aladdin, China) solution (25 mg/mL), and 2 mL of K@BMT dispersion (50 mg/mL). After the addition of all aqueous components, the mixture was kept under constant stirring at 500 rpm for 30 min. During this process, the aqueous phase was sheared and dispersed into fine

droplets within the oil phase, forming a stable water-in-oil (W/O) primary emulsion under the stabilization of the emulsifier, with K@BMT core encapsulated inside the aqueous droplets. After the emulsification process, the three-neck flask was removed from the 45 °C water bath and immediately placed in an ice-water mixture (0-4 °C) for rapid cooling. Once the emulsion system had completely cooled, 2 mL of glutaraldehyde aqueous solution was added to initiate crosslinking. The reaction was maintained under continuous stirring at 500 rpm for 20 min in the ice bath. Upon completion, the entire emulsion was transferred into centrifuge tubes and centrifuged at 5000 rpm for 10-15 min to allow the hydrogel microspheres to precipitate. The supernatant was carefully decanted, and an appropriate volume of acetone (Aladdin, China) was added to the precipitate, which was ultrasonically dispersed to redisperse the microspheres, followed by centrifugation at 5000 rpm for 10 min, after which the supernatant was discarded. This washing step was repeated three times to completely remove residual paraffin oil and organic solvents. The precipitate was subsequently washed three additional times with ultrapure water to remove residual acetone, unreacted crosslinker, and other water-soluble impurities. Finally, the purified hydrogel microsphere precipitate was redispersed in 10 mL of perbenzoic acid (PBA, BenchChem, USA) solution and transferred into sample vials. The samples were stored at 4 °C for later use. Thus, thermosensitive hydrogel microspheres loaded with self-assembled nanoparticles were successfully obtained.

1.17. Characterization and Performance Verification of Nanomedicine

The morphology and elemental mappings of K@BMT@HM were tested via scanning electron microscopy (SEM, MIRA4, TESCAN, Czech). To test the lubrication performance of K@BMT@HM, microsphere powder was evenly fixed onto a flat substrate, ensuring that the sample surface was as smooth as possible without obvious large-particle protrusions. The counterpart ball was mounted on the upper fixture of the tribometer (UMT Tribolab, Bruker, USA), and the prepared microsphere sample was secured beneath it. The relative position was adjusted to ensure that the counterpart ball was vertically aligned with the center of the sample surface. After setting the experimental parameters, the motor was started to drive the counterpart ball in a reciprocating motion across the sample surface. During the test, the tribometer continuously and automatically recorded the coefficient of friction (COF) in real time. To evaluate the photothermal effect of K@BMT@HM *in vivo*, irradiation of rat joints was performed using an 808-nm laser at the power density of 2 W/cm^2 for a duration of 10 min. The temperature of the joints was recorded at 2-min intervals using thermal camera and thermal images were captured.

1.18. Establishment of OA rat models and intra-articular injection

20 SPF-grade male Wistar rats (weight 250 g) were randomly divided into 4 groups, including SHAM, VEH, K@BMT, and K@BMT-L group. The rats of SHAM group were executed the sham operation as opening the joint capsule merely, while the rats of VEH, K@BMT, and K@BMT-L group were established as OA models by anterior cruciate ligament transection (ACLT) and destabilization of the medial meniscus (DMM) after anesthetized with intraperitoneal injection of pentobarbital sodium. Rats were injected intramuscularly with benzylpenicillin potassium to prevent infection for 3 consecutive days after surgery. The rats of VEH, K@BMT, and K@BMT-L group were enforced to run on a rodent treadmill for 1 h per day, then the OA models were built 4 weeks later. After the OA rat models were established, the rats of VEH, K@BMT, and K@BMT-L group were carried out intra-articular injections, and intervened with vehicle (physiological saline), K@BMT, and K@BMT under 808 nm laser (10 min a time) twice a week for 12 weeks consecutively.

1.19. Functional tests

Hot plate test was applied to assess thermal nociceptive response in the knee joint. Rats from each group were placed on a hot plate apparatus (Ugo Basile, Italy) maintained at 55 °C, and the latency to hindlimb responses such as shaking or licking was recorded. To prevent tissue damage, animals were immediately removed if the response latency exceeded 30 s. Each rat was tested three times with adequate intervals between trials. Weight bearing test was used to evaluate pain-related changes in hindlimb load distribution. Rats from each group were positioned with their bilateral hindlimbs on a bipedal balance pain tester (Bioseb, France), and the static load of each limb was recorded over a fixed duration (9 s). The difference in weight distribution between the right and left hindlimbs was calculated as an indicator of altered weight bearing due to joint discomfort or pain. Each animal was measured three times with consistent positioning and intervals between measurements. Behavioral assessments were performed by investigators blinded to group assignments.

1.20. Fluorescence imaging (FI) analysis

The levels of ROS in rat knee joints at different stages were detected by FI. Specifically, intra-articular injections of the CellROX probe were administered into the knee joints, and the corresponding fluorescence intensities were subsequently measured using the IVIS Spectrum system.

1.21. Micro Computed Tomography (Micro-CT) imaging analysis

The rat knee joints were observed through micro-CT imaging and 3D reconstruction. The rats of each group were anesthetized with intraperitoneal injection of pentobarbital sodium and scanned with a Micro-CT instrument (SkyScan 1276, Bruker, Germany), while the results were analyzed via Data Viewer, CTAn and CTVol in order to calculate total volume of osteophytes, bone trabecular volume

per total volume (BV/TV) and trabecular bone pattern factor (Tb. pf).

1.22. Magnetic resonance imaging (MRI) analysis

To evaluate the intra-articular targeting and retention of Mn-containing nanomedicine, rats received intra-articular injection of K@BMT@HM or control formulations into the knee joint. After an uptake interval prior to imaging, animals were anesthetized and positioned in a small animal MRI scanner. T1-weighted MRI was acquired on a high-field MRI system (3.0 T small animal MRI, Germany) using a spin-echo or gradient-echo sequence optimized for rat knee imaging. Imaging parameters included repetition time (TR), echo time (TE), field of view (FOV), slice thickness, and matrix size appropriate for joint anatomy. The paramagnetic manganese ions in the K@BMT@HM nanomedicine served as an intrinsic T1 contrast agent to generate positive signal enhancement on T1-weighted images. MRI data were reconstructed and analyzed using the manufacturer's software to assess signal enhancement and spatial distribution of the injected nanomedicine within the joint.

1.23. Hematoxylin–eosin (HE) staining assay

Paraffin-embedded rat knee joint sections were deparaffinized in xylene and rehydrated through a graded ethanol series (100%, 95%, 85%, 75%) to distilled water. Sections were stained with hematoxylin (Solarbio, China) and then counterstained with eosin (Solarbio, China). After dehydration through increasing ethanol concentrations and clearing, sections were imaged using a digital slide scanner. HE staining highlights nuclei and general tissue morphology in joint sections, serving as a routine histological assessment method for morphology evaluation.

1.24. Alcian blue (AB) staining assay

Chondrocytes and rat knee joint sections were stained with 1% AB solution (Solarbio, China) to demonstrate acidic proteoglycans and glycosaminoglycans. After staining, samples were rinsed with running tap water followed by distilled water. Tissue sections were dehydrated, cleared, and scanned with a digital slide scanner, and cell climbing sheets were observed under a microscope.

1.25. Safranin O–fast green (SF) staining assay

Paraffin-embedded rat knee joint sections were deparaffinized and stained sequentially with Weigert's iron hematoxylin (Solarbio, China), fast green (Solarbio, China), and Safranin O (Solarbio, China). After brief differentiation and rinses, sections were dehydrated through graded ethanol and cleared, then scanned with a digital slide scanner. For chondrocytes, Safranin O (SO) staining was performed without fast green counterstaining, and cell climbing sheets were examined under a microscope.

1.26. Immunohistochemistry (IHC) staining assay

The paraffin slices were hydrated with ethanol orderly for 3 min after dewaxing and washed with PBST buffer 3 times. The antigens of the tissue sections were repaired with citrate antigen retrieval solution at 95°C for 10 min. Next, the sections were incubated using endogenous peroxidase blocker (Absin, China) for 20 min away from light, then the sections were blocked with normal goat serum (Absin, China) for 60 min at room temperature and rinsed several times with PBST buffer. The sections of explants and joints were incubated with the designated primary antibody at 4 °C for 6 h and pumped with the HRP enzyme-linked secondary antibody (Absin, China) at room temperature for 1 h away from light. The sections were added to DAB chromogen (Absin, China) prepared freshly and incubated at room temperature for 5 min. Then, the sections were incubated with hematoxylin (Absin, China) for 3 min at room temperature and washed with running water. The above slices, sealed with resin, were scanned into images through digital slide scanner. The information of primary antibody applied for immunohistochemistry assay were shown in Supplementary Information (Table S3).

1.27. OA Research Society International (OARSI) score

The OARSI scoring system was used to evaluate histopathological changes in the knee joint. Articular cartilage damage and osteophyte formation were scored separately in the medial and lateral femoral condyles and tibial plateaus, and these regional scores were summed to obtain a subtotal. In addition, the overall severity of cartilage degeneration across the joint was assessed to derive a global score. The final OARSI score was calculated as the sum of these components, with higher scores indicating more severe osteoarthritic changes (maximum possible score 30).

1.28. Blood test

After euthanasia, blood was collected from rats using anticoagulant tubes for whole blood analysis and clot-activating tubes for serum collection. Whole blood was subjected to routine hematological examination using an automatic hematology analyzer to measure parameters to evaluate general blood cell status. Serum was separated by centrifugation and used for biochemical assessment of liver and kidney function. In addition, serum manganese (Mn) concentration was measured as a trace element indicator to evaluate systemic Mn levels in serum.

1.29. Biosafety analysis

The organs of rats, including hearts, livers, kidneys, lungs and spleens, were fixed with 4% paraformaldehyde for 48 h and embedded in paraffin and cut into 5 μm-thick sections. Next, HE staining was used to evaluate whether the organs were damaged after the intervention of K@BMT, and K@BMT under 808 nm laser, while the sections were scanned into images through digital slide scanner.

2. Supplementary figures

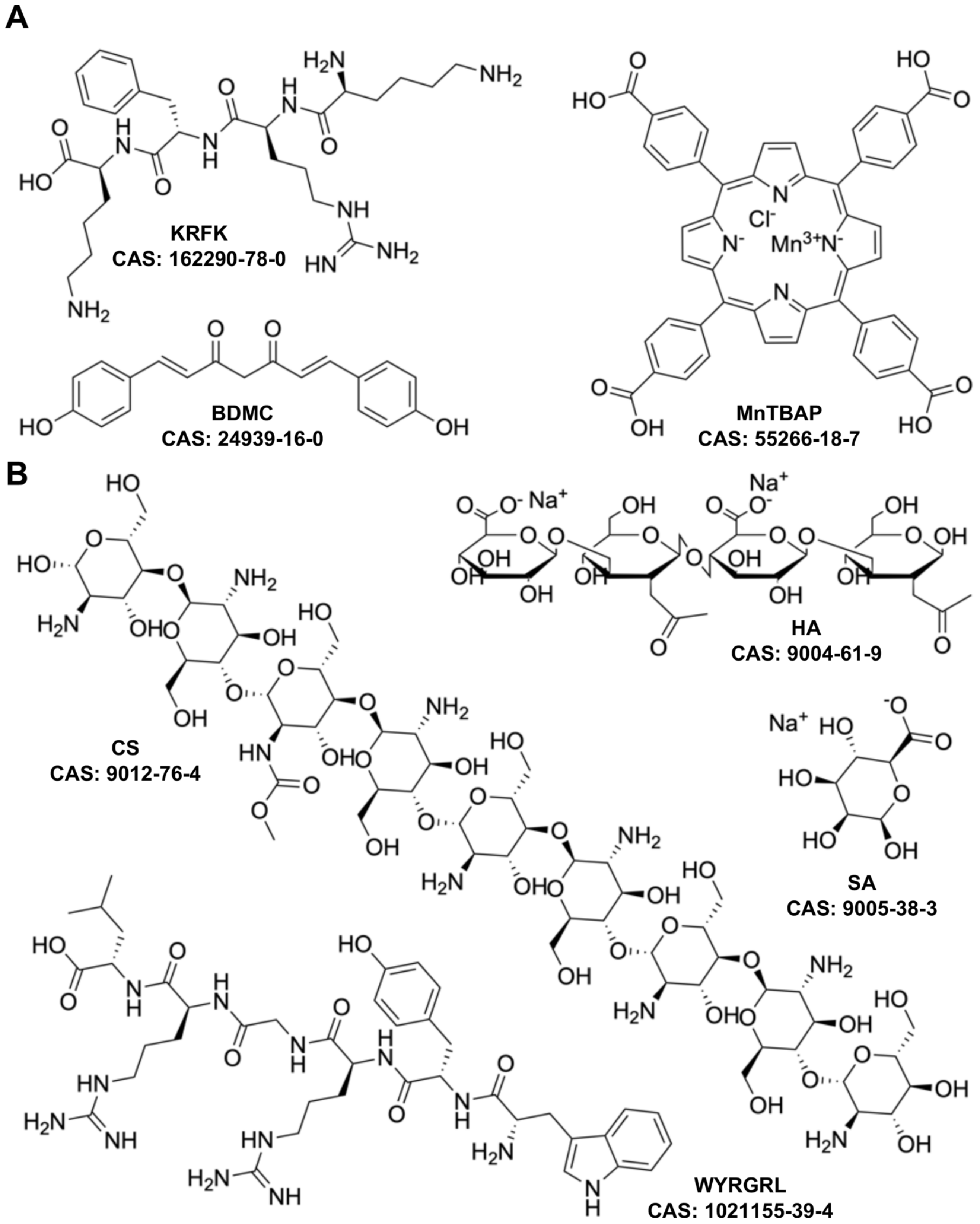

Figure S1. (A) Chemical structures of KRFK, BDMC, and MnTBAP monomers. **(B)** Chemical structures of CS, SA, HA, and WYRGRL monomers.

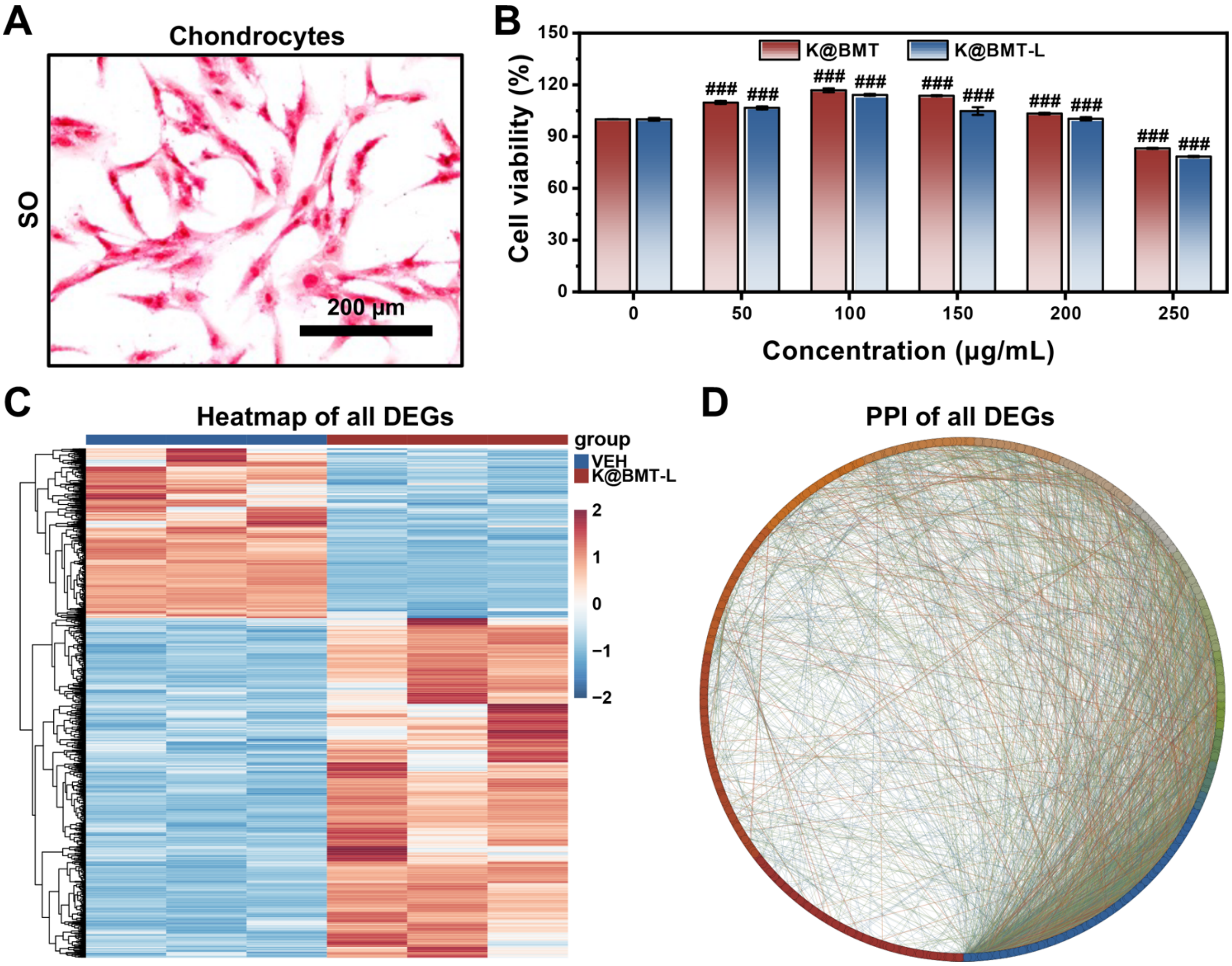

Figure S2. (A) SO staining images of chondrocytes (n = 3). **(B)** Cell viability of chondrocytes determined by CCK-8 (n = 3). **(C)** Heatmap of all DEGs of chondrocytes. **(D)** PPI network of all DEGs of chondrocytes. All data are presented as the mean ± SD ($^{\#}$Compared with "0 μg/mL" group, $^{\#\#\#}P < 0.001$).

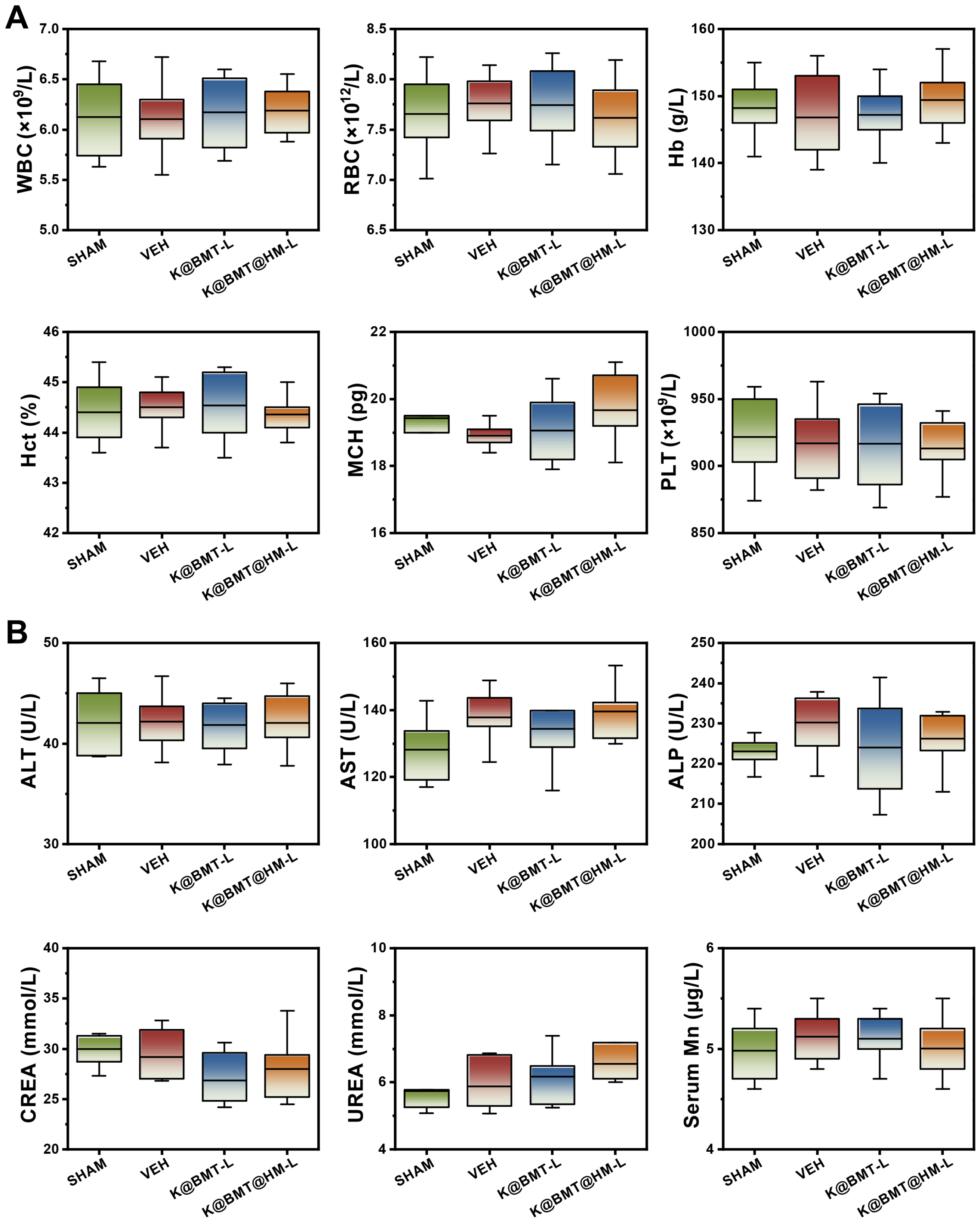

Figure S3. (A) Blood routine examination for WBC, RBC, Hb, Hct, MCH and PLT in rats (n = 5). **(B)** Blood biochemical examination for ALT, AST, ALP, CREA, UREA, and serum Mn in rats (n = 5).

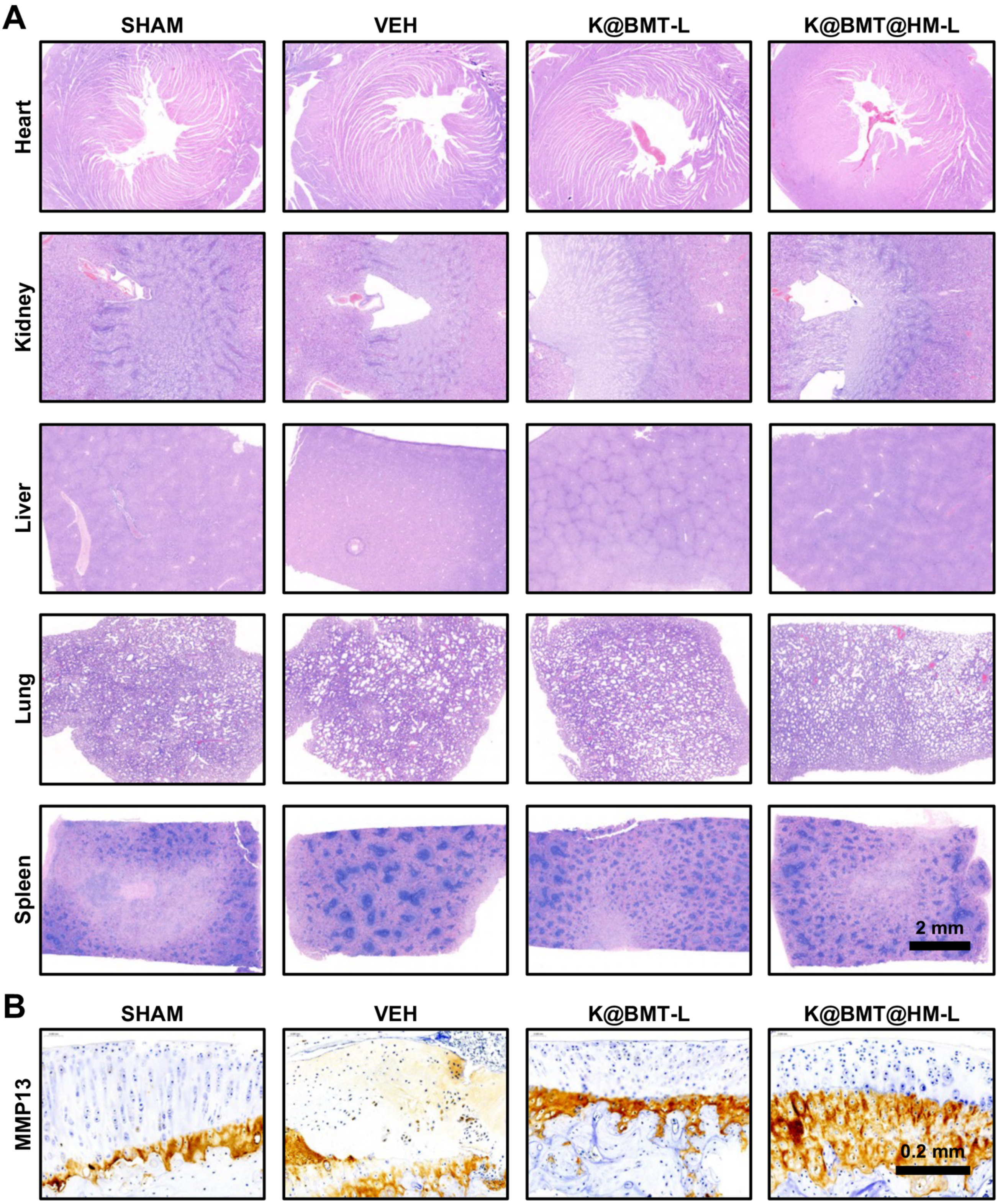

Figure S4. (A) Organ pathological assessment of hearts, kidneys, livers, lungs, and spleens from rats (n = 5). **(B)** IHC staining images of rat knee joints for MMP13 (n = 5).

3. Supplementary tables

Table. S1. The information of primary antibody applied for WB analysis.

Protein	Producer	Application	Molecular Weight
PRG4	ABclonal, China	1:1000	150 kDa
SOX9	Abcam, USA	1:1000	70 kDa
ACAN	Abcam, USA	1:1000	110 kDa
COL2A1	Abcam, USA	1:1000	36 kDa
ADAMTS5	Abcam, USA	1:500	73 kDa
MMP13	Abcam, USA	1:500	54 kDa
SQSTM1	ABclonal, China	1:2000	62 kDa
ATG5	ABclonal, China	1:1000	55 kDa
ATG16L1	ABclonal, China	1:1000	68 kDa
LC3B-I/II	Cell Signaling Technology, USA	1:1000	14/16 kDa
KEAP1	ABclonal, China	1:1000	60 kDa
NFE2L2	Abcam, USA	1:1000	85 kDa
NOX4	ABclonal, China	1:500	67 kDa
PKC	ABclonal, China	1:1000	76 kDa
HMOX1	ABclonal, China	1:1000	33 kDa
NQO1	ABclonal, China	1:1000	31 kDa
GCLC	ABclonal, China	1:1000	72 kDa
SOD2	ABclonal, China	1:1000	25 kDa
GPX1	ABclonal, China	1:1000	22 kDa
CAT	ABclonal, China	1:1000	60 kDa
TGFB1	ABclonal, China	1:1000	25 kDa
TGFB3	ABclonal, China	1:500	60 kDa
TGFBR1	ABclonal, China	1:1000	56 kDa
TGFBR2	ABclonal, China	1:1000	90 kDa
SMAD2	ABclonal, China	1:1000	60 kDa
P-SMAD2	ABclonal, China	1:1000	60 kDa
SMAD3	ABclonal, China	1:1000	52 kDa

P-SMAD3	ABclonal, China	1:1000	52 kDa
RELA	ABclonal, China	1:1000	65 kDa
P-RELA	Cell Signaling Technology, USA	1:1000	65 kDa
NFKBIA	ABclonal, China	1:1000	40 kDa
P-NFKBIA	ABclonal, China	1:1000	39 kDa
TP53	ABclonal, China	1:1000	53 kDa
P-TP53	ABclonal, China	1:1000	53 kDa
BAX	ABclonal, China	1:1000	21 kDa
BCL2	ABclonal, China	1:1000	26 kDa
CASP3	ABclonal, China	1:1000	32 kDa
C-CASP3	Cell Signaling Technology, USA	1:1000	17 kDa
PARP1	ABclonal, China	1:1000	89 kDa
C-PARP1	Cell Signaling Technology, USA	1:1000	27 kDa
ACTB	ABclonal, China	1:5000	42 kDa
VCL	ABclonal, China	1:10000	124 kDa
TUBA	ABclonal, China	1:1000	55 kDa

Table. S2. The information of primary antibody applied for IF staining.

Protein	Producer	Application
ACAN	Thermo Fisher Scientific, USA	1:500
COL2A1	Abcam, USA	1:200
SQSTM1	Abcam, USA	1:100
NFE2L2	Cell Signaling Technology, USA	1:400
P-RELA	Cell Signaling Technology, USA	1:500
CYCS	Abcam, USA	1:100

Table. S3. The information of primary antibody applied for IHC staining.

Protein	Producer	Application
ACAN	Thermo Fisher Scientific, USA	1:50
COL2A1	Abcam, USA	1:100
MMP13	Abcam, USA	1:50
